# Knowledge gaps and research priorities in adult veno-arterial extracorporeal membrane oxygenation: a scoping review

**DOI:** 10.1186/s40635-022-00478-z

**Published:** 2022-11-25

**Authors:** Senta Jorinde Raasveld, Carolien Volleman, Alain Combes, Lars Mikael Broman, Fabio Silvio Taccone, Elma Peters, Sanne ten Berg, Charissa E. van den Brom, Holger Thiele, Roberto Lorusso, José P. S. Henriques, Alexander P. J. Vlaar

**Affiliations:** 1 Department of Critical Care, Amsterdam University Medical Centers, Location Academic Medical Center, Amsterdam, The Netherlands; 2 Institute of Cardiometabolism and Nutrition, Sorbonne Université, INSERM, UMRS1166-ICAN, Paris, France; 3 Service de Médicine Intensive-Réanimation, Institut de Cardiologie, APHP Sorbonne Hospital Pitié-Salpêtrière, Paris, France; 4 Department of Physiology and Pharmacology, Karolinska Institutet, Stockholm, Sweden; 5 Perioperative Medicine and Intensive Care, Karolinska Institutet, Stockholm, Sweden; 6 Department of Intensive Care, Université Libre de Bruxelles, Hôpital Universitaire de Bruxelles (HUB), Brussels, Belgium; 7 Heart Center, Department of Cardiology, Amsterdam Cardiovascular Sciences, Amsterdam UMC, University of Amsterdam, Amsterdam, The Netherlands; 8 Laboratory of Experimental Intensive Care and Anesthesiology, Amsterdam UMC, University of Amsterdam, Amsterdam, The Netherlands; 9 Heart Center Leipzig at University of Leipzig and Leipzig Heart Science GmbH, Leipzig, Germany; 10 Department of Cardio-Thoracic Surgery, Heart and Vascular Centre, Maastricht University Medical Centre (MUMC), Cardiovascular Research Institute Maastricht (CARIM), Maastricht, The Netherlands; 11 Department of Anesthesiology, Amsterdam UMC, VU University, Amsterdam, The Netherlands

**Keywords:** Veno-arterial extracorporeal membrane oxygenation, Extracorporeal cardiopulmonary resuscitation, Research gaps

## Abstract

**Purpose:**

This scoping review aims to identify and describe knowledge gaps and research priorities in veno-arterial extracorporeal membrane oxygenation (VA-ECMO).

**Methods:**

An expert panel was recruited consisting of eight international experts from different backgrounds. First, a list of priority topics was made. Second, the panel developed structured questions using population, intervention, comparison and outcomes (PICO) format. All PICOs were scored and prioritized. For every selected PICO, a structured literature search was performed.

**Results:**

After an initial list of 49 topics, eight were scored as high-priority. For most of these selected topics, current literature is limited to observational studies, mainly consisting of retrospective cohorts. Only for ECPR and anticoagulation, randomized controlled trials (RCTs) have been performed or are ongoing. Per topic, a summary of the literature is stated including recommendations for further research.

**Conclusions:**

This scoping review identifies and presents an overview of knowledge gaps and research priorities in VA-ECMO. Current literature is mostly limited to observational studies, although with increasing attention for this patient population, more RCTs are finishing or ongoing. Translational research, from preclinical trials to high-quality or randomized controlled trials, is important to improve the standard practices in this critically ill patient population.

**Take-home message**

This scoping review identifies and presents an overview of research gaps and priorities in VA-ECMO. Translational research, from preclinical trials to high-quality or randomized controlled trials, is important to improve the standard practices in this critically ill patient population.

**Supplementary Information:**

The online version contains supplementary material available at 10.1186/s40635-022-00478-z.

## Introduction

Veno-arterial extracorporeal membrane oxygenation (VA-ECMO) is a mechanical circulatory support (MCS) used for refractory cardio-circulatory failure, in case conventional therapies prove insufficient [1]. The past decades, the use and range of indications for VA-ECMO has been increasing worldwide. In 2021, almost 5000 VA-ECMO runs were recorded in the ELSO registry, of which 48% survived [2]. Despite the promising role of VA-ECMO as a cornerstone supportive treatment, complication rates remain high and should be better evaluated in daily practice. Available guidelines are mainly based on expert opinion, resulting in a high variance in local protocols worldwide. It is important to identify topics which require further attention in this complex, critically ill patient population. Therefore, the aim of this study is to identify and describe research gaps in VA-ECMO, and to form recommendations for future research.

## Methods

This scoping review was performed in several steps. Firstly, an expert panel was developed consisting of eight international experts with research lines and backgrounds in different medical specialties involved in VA-ECMO practices (Additional file 1 p.2). Secondly, an initial list of topics was developed by JR, CV and AV. The expert panel was invited to submit additional topics to this list and give feedback on the topics stated. Thirdly, per topic, structured questions using population, intervention, comparison and outcomes (PICO) format were created. Fourthly, the PICOs were prioritized by rating the importance of every PICO on a scale of 0–10 using a cloud-based survey tool. The eight PICOs with the highest ratings were considered as highest priority. Fifthly, search strategies were developed for every selected PICO, whereafter searches in MEDLINE, EMBASE, Web of Science, Google Scholar, and Cochrane databases were performed up to September 2022, along with trial databases (clinicaltrials.gov, ISRCTN) to identify in-progress trials. Per topic, the search results were screened by two experts (Additional file 1 p. 9–40).

## Results

**Fig. 1:**
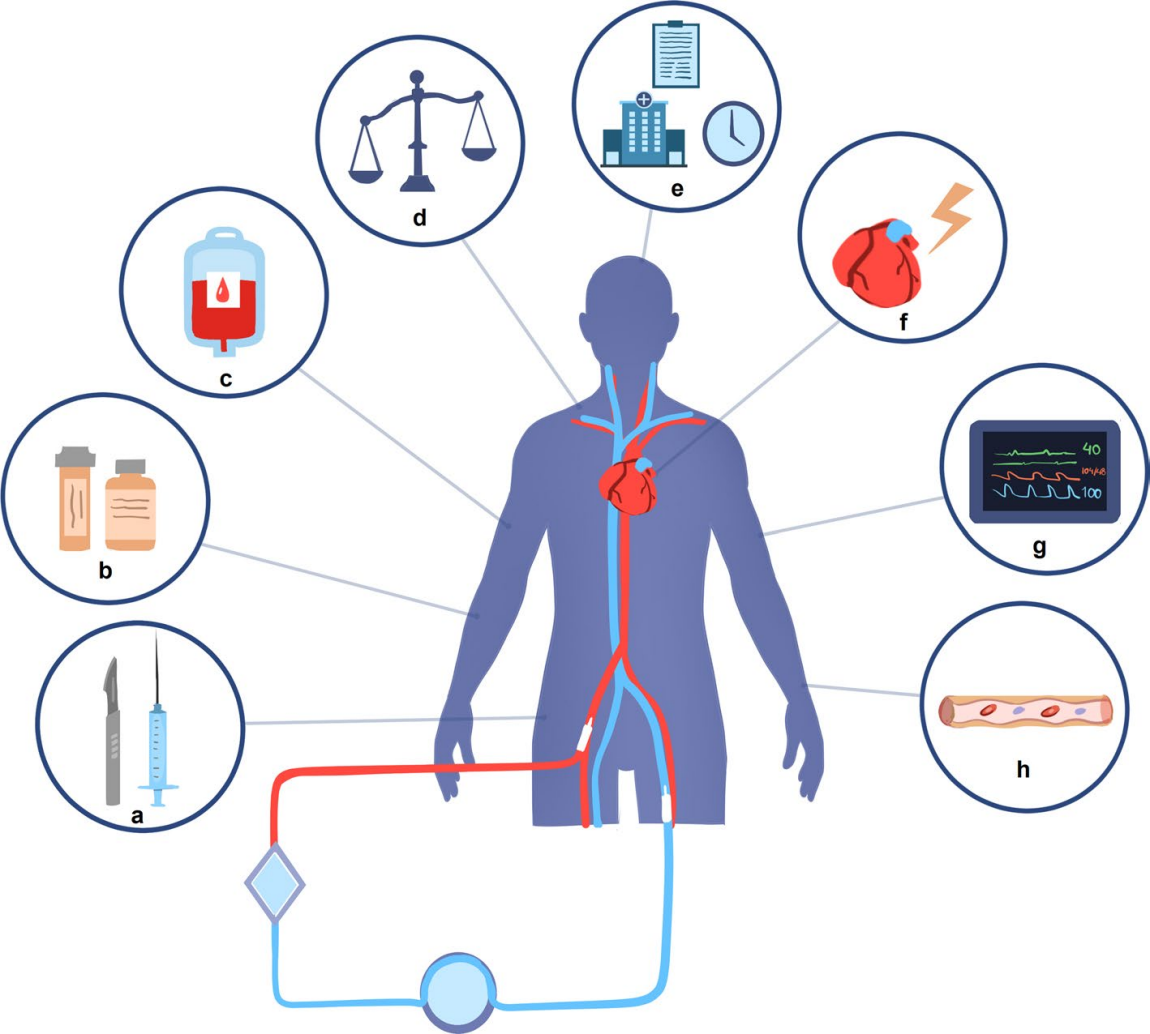
Priority topics further elaborated in this review. From left to right: **a** percutaneous versus surgical methods of cannulation, **b** anticoagulant therapies, **c** blood transfusion regimen, **d** daily therapy goals, **e** ECPR selection criteria, **f** cardiogenic shock, **g** optimal balance of blood pressure and vasoactive medication, **h** endothelial activation and damage. © Myrthe Raasveld

The initial list of 49 topics can be found in the Additional file 1 (p. 3–8). After rating the degree of importance, eight topics were selected as depicted in Table 1 and shown in Fig. 1.

**Table 1:** Priority topics and corresponding PICOs

No.	Topic	Population	Intervention	Control	Outcome	Sub-topics
1	Cardiogenic shock: definition, degree and timing of cardiogenic shock as VA-ECMO indication	Patients suffering cardiogenic shock^1^	Early in course	Refractory cardiogenic shock	Shock reversal, in-hospital mortality	–
2	Selection criteria for ECPR	Patients suffering cardiac arrest	ECPR (VA-ECMO)	Conventional cardiopulmonary resuscitation	In-hospital mortality, length-of-stay	Patient age Time-to-ECMO Location of insertion: in-hospital/on-site
3	ECMO connecting: percutaneous versus surgical methods	Patients undergoing cannulation for VA-ECMO	Percutaneous cannulation	Surgical cannulation	Hemorrhage, in-hospital mortality	–
4	Monitoring: daily therapy goals	Patients supported with VA-ECMO	Daily goals	Standard care	In-hospital mortality	Fluid balance, min/max Reducing blood flow Reducing sedatives
5	Monitoring: optimal balance of blood pressure & vasoactive medication	Patients supported with VA-ECMO	Accepting a lower MAP (< 65 mmHg)	Standard MAP (≥ 65 mmHg)	In-hospital mortality	–
6	Blood transfusion regimen	Patients supported with VA-ECMO	Restrictive regimen (dependent of blood product)	Liberal regimen (dependent of blood product)	In-hospital mortality	Red blood cells Platelets Plasma
7	Anticoagulant therapies	Patients supported with VA-ECMO	Non-heparin anticoagulant therapy	Continuous systemic heparin	In-hospital mortality	LMWH Bivalirudin DTIs
8	Complications: endothelial activation and damage	Patients supported with VA-ECMO	Monitoring endothelial damage and activation markers	No additional markers/monitoring	In-hospital mortality	ICAM-1 VCAM Syndecam-1

*DTI* direct thrombin inhibitors, *ICAM-1* intercellular adhesion molecule 1, *MAP* mean arterial pressure, *ROSC* return of spontaneous circulation, *VA-ECMO* veno-arterial extracorporeal membrane oxygenation, *VCAM* vascular cell adhesion molecule

^1^Defining early in course and refractory as INTERMACS level 2 (“sliding on inotropes”) and level 1 (“crash and burn”), respectively

### Part I: indication for ECMO

#### #1 Cardiogenic shock: definition, degree and timing of cardiogenic shock as VA-ECMO indication

##### Timing of ECMO

Clinical benefits of VA-ECMO for cardiogenic shock (CS) due to acute myocardial infarction (AMI) have been demonstrated in observational studies [3]. For defining the degree of severity of cardiogenic shock, different classification systems are available. Most commonly used classifications include the INTERMACS (Interagency Registry for Mechanically Assisted Circulatory Support) and Society for Cardiovascular Angiography and Interventions (SCAI). VA-ECMO may play a role in either INTERMACS’ level 1 and 2 (1: “*crash and burn*” vs. 2:“early” phase, “*sliding on inotropes*”). Initiation is usually employed for patients refractory to usual resuscitative techniques (i.e., inotropes and vasopressors [4]. Guidelines recommend that MCS, including VA-ECMO, may be considered in patients with either any type of CS (Heart Failure Guidelines [5]) or due to acute coronary syndrome (ACS, European Guidelines [6]). However, there is no consensus on optimal timing. ‘*The sooner the better*’ seems likely to assume, but its relation with the timing of reperfusion, and to the risk benefit of any MCS device remains unclear.

##### Before or after percutaneous coronary intervention (PCI)

In patients with CS from different etiologies, shorter time from shock onset to ECMO insertion is associated with lower risk of mortality in observational studies [7]. In selected patients with CS due to AMI undergoing PCI, early ECMO initiation prior to PCI resulted in better short- and long-term outcomes, even though this resulted in a longer door-to-balloon time [7–10]. However, respective studies included small numbers of patients, of whom up to 60% experienced a cardiac arrest. In a single cohort of AMI-CS patients that excluded post-cardiac arrest patients, no association between time of CS onset to VA-ECMO start time and 6-month survival was found [11]. Moreover, no conclusion can yet be drawn regarding which MCS could be most beneficial. Available studies show divergent results, whereas crucial variables as timing and severity of disease may be insufficiently corrected for RCTs on this topic are lacking.

##### Extracorporeal cardiopulmonary resuscitation (ECPR)

In case of refractory cardiac arrest, ECPR combined with intra-arrest PCI leads to better outcomes in terms of survival as shown in the highly selected ARREST trial [12]. In the larger PRAGUE-OHCA trial such a mortality benefit could not be observed, although it does suggest a favorable neurological outcome in patients receiving ECPR [13]. Contrarily, observational studies suggest that a delay in the start of ECPR is an independent predictor of poor neurological outcome and should ideally be minimalized to < 40 min [14].

##### Future research

Timing of VA-ECMO in CS needs further attention. In line with this, we would recommend to also focus on the simultaneous or consecutive use of alternative MCS such as Impella (“ECPELLA”).

#### #2 Patient selection criteria for ECPR

Patient selection for ECPR is a significant challenge for clinicians: on one hand, stringent selection criteria for ECPR would result in a higher proportion of patients with favorable outcome, however, this would also significantly limit its use in victims of refractory cardiac arrest [15]. Existing literature on ECPR effectiveness is mainly limited to cohort studies, with recently first RCTs published (Table 2) [12, 13, 16, 17].

**Table 2:** Selected overview of eligibility criteria in RCTs performed on ECPR

*Eligibility criteria*	*ARREST trial* *Yannopoulos *et al	*Prague-OHCA trial* *Behlohlavek *et al	*INCEPTION trial* *Bol *et al
*Age*	≥ 18 and ≤ 75 years	≥ 18 and ≤ 65 years	≥ 18 and ≤ 70 years
*Duration of ongoing resuscitation without obtaining ROSC*	No ROSC within the first 3 shocks Estimated transfer time to ED < 30 min	≥ 5 min of advanced cardiovascular life support without ROSC	No ROSC ≤ 15 min Expected initiation of cannulation < 60 min. after arrest
*Location of insertion*	In-hospital	In-hospital	In-hospital
*Initial rhythm*	VT/VF	OHCA with presumed cardiac cause (shockable and non-shockable rhythms)	VT/VF

*ED* emergency department, *ROSC* return of spontaneous circulation, *VT* ventricular tachycardia, *VF* ventricular fibrillation

##### Age and patients’ characteristics

Latest ELSO interim guidelines suggest a maximum age of 70 years for ECPR [18]. However, a large retrospective study found that in case of a low-flow state < 60 min, ECPR survival rates were independent of age, suggesting that some elderly patients could be still be considered [19]. At the time of ECPR assessment, patient’s medical history, laboratory values and severity scores are often unknown or difficult to obtain and can rarely be used into the decisional algorithm. Whether only patients with an initial shockable rhythm should be treated remains a matter of debate, as acceptable survival rates have been reported also for non-shockable rhythm, in particular pulseless activity [20, 21].

##### Time-to-ECMO and location of ECMO insertion

Time-to-ECMO is associated with neurological outcome and survival. The ELSO advises early assessment for ECPR and time from arrest to ECMO (i.e., “low-flow interval”) to be < 60 min [18]. Different cut-offs of no-flow or low-flow intervals have been evaluated in observational studies, varying from 30 to 60 min. Importantly, the longer the low-flow interval, the higher the difference in outcome between ECPR and conventional cardiopulmonary resuscitation (CPR) treated patients, whereas nearly 20% of patients undergoing ECPR with a time-to-ECMO > 60 min would eventually still experience favorable neurological outcome [22].

To reduce the low-flow time, which remains of approximately one hour even in RCTs [12, 13], there are two main strategies: (a) expedited transport to the hospital to reduce the time to hospital arrival [16]; or (b) pre-hospital ECMO implantation [23]. Currently, a stepped-wedge designed trial comparing on-scene initiation of ECPR and conventional CPR, is ongoing in the Netherlands (NCT04620070).

##### Future research

As ECPR is also dependent of country-specific logistics and infrastructure, identifying optimal patient selection criteria is an essential step to further evaluate the role of ECPR.

### Part II: ECMO cannulation

#### #3 ECMO connecting: percutaneous vs surgical cannulation methods

Multiple cannulation techniques have been widely accepted for VA-ECMO. In case of post-cardiotomy patients, central placement of the cannulas might be considered, although recent data showed higher mortality rate as compared to peripheral access in such a setting [24, 25]. In non-cardiotomy patients, peripheral cannulation, either via percutaneous or surgical placement, is preferred due to the speed and easy accessibility [1]. Moreover, next to the indication, location (in-hospital or on-scene) can play a role in choice of cannulation technique. Despite their practical differences, limited data are available on the impact of placement methods on patients’ outcomes.

##### Percutaneous vs surgical cannulation

A recent analysis of the ELSO Registry including 12,592 patients receiving VA-ECMO showed a decreased in-hospital mortality in favor of percutaneous cannulation when compared to the surgical group [26]. This finding was supported by a propensity-score matched analysis comparing complication rates and survival in surgical versus percutaneous peripheral cannulation [27]. This can be explained by a lower degree of cannulation site bleeding and systematic infection in case of percutaneous cannulation [26]. However, percutaneous cannulation was found to be associated with an increased rate of vascular complications after decannulation, for which further attention is needed [27].

Both surgical as peripheral cannulation bring the risk of limb ischemia. As a result, following femoral cannulation, the adequacy of limb perfusion and absence of ischemia should be carried out by the use of near-infrared spectroscopy (NIRS). If NIRS is > 50–60% at both legs, a distal perfusion catheter (DPC) might be, theoretically, not necessary. However, distal limb perfusion is always recommended to avoid late intervention in case of leg ischemia, with the occurrence of potential irreversible injury. Either in case of larger cannulas (19–21 Fr), a NIRS-value < 50–60%, or a difference over 20% between the arterial and venous cannulated leg, DPCs are recommended [28].

##### Future research

Less invasive percutaneous approach appears to be favorable in terms of complications and patient outcome. However, this does not take into account the rate of cannulation failure, which may be higher using percutaneous methods. With the rising interest of ECPR, complication rates between different locations of cannulation (in-hospital vs. on-scene) should also be taken into account. Also, decannulation differs between the two methods, leading to different complication profiles. Future studies should focus on preventing cannulation failure and improving decannulation care, such as removal techniques, in a prospective setting.

### Part III: ECMO support care

#### #4 Monitoring: daily therapy goals

##### Reducing blood flow

ELSO guidelines state the ideal situation in patients on VA-ECMO would be to decrease blood flow until the arterial pulse pressure is a minimum of 10 mmHg or to provide adequate support, according to other parameters of organ perfusion [1]. As over time the native cardiac function is expected to improve, there is rationale for evaluating on a daily basis whether blood flow can be reduced. However, few data have been reported about the course of ECMO blood flow and its manipulation over time in this setting.

##### Fluid balance

ECMO blood flow and patient’s volume status are intertwined, as on one hand sufficient intravascular volume is required to ensure adequate blood flow and organ perfusion, while on the other hand volume overload has to be prevented, despite the high occurrence rate of blood transfusions and the occurrence of acute kidney injury in these patients. Retrospective studies in VA-ECMO patients also showed a correlation between a higher fluid balance and mortality [29–31]. However, no recommendations regarding optimal fluid balances are described in the latest ELSO guideline, and no high-quality data on the optimal fluid strategy are available for such patients [32].

##### Reducing sedatives

Factors influencing sedative and analgesic management include additional treatments, such as the application of targeted temperature management, but also cannula related discomfort, and a possible change in drug pharmacokinetics due to the ECMO system (i.e., drug absorption) [33]. For sedation, it is commonly advised to provide light anesthesia in the first 24 h and then adjust therapy to relieve patient’s anxiety and discomfort, but still allowing a daily repeated neurological examination [1]. For some indications, such as bridge to lung transplant, awake support is also a possibility. No hard recommendations can be made based on current literature regarding the use of paralytics, whereas different factors should be taken into account (i.e., need for controlled mechanical ventilation, physical therapy). With regard to sedative management, different observational studies reported comparisons of different anesthetics and analgesics used, but otherwise literature is scarce.

##### Future research

In different critically ill patient populations, current studies focus more and more on how to prevent or treat fluid overload, for example using lung ultrasound. Prospective studies VA-ECMO patients, focusing on visualizing and evaluating fluid status and blood flow, are needed.

### #5 Monitoring: optimal balance of blood pressure and vasoactive medication, “less is more?”

In all-cause shock patients, a target mean arterial pressure (MAP) of above 65 mm Hg is being aimed for. This is mostly based on settings of sepsis, in which below this threshold autoregulation fails and tissue perfusion becomes dependent on the MAP’s driving pressure [34]. Current MAP targets in other shock etiologies are based on the same principles, despite having a different pathophysiology. In cardiogenic shock, no clear MAP targets can be set due to limited supporting data [35].

Similarly, in (VA-)ECMO the optimal MAP target remains unknown. ELSO guidelines lack recommendations, except for when ‘left ventricle overloading’ occurs, in which it is recommended to reduce the MAP to the lowest acceptable value [36]. In case of ECPR, ELSO guidelines recommend a MAP between 60 and 80 mmHg [18]. Interestingly, a recent RCT studying blood pressure targets in comatose OHCA patients found equal survival and neurological outcomes in the low- (63 mm Hg) versus high-target group (77 mm Hg) [37]. Theoretically, as the heart has to eject against a continuous blood flow generated by the ECMO device, a lower MAP seems favorable as it reduces afterload, and therefore decreases myocardial oxygen demand, possibly optimizing native cardiac output in the already failing heart [38–40].

Currently, only one study addresses the question of optimal MAP targeting in adult patients on ECMO. In this retrospective observational study of 116 patients, a higher average MAP was associated with survival to discharge [38]. Furthermore, in patients with a lower MAP, a higher incidence of kidney injury was found. However, a lower MAP might also be the result of other factors contributing to the poor prognosis, rather than the cause. Due to its retrospective character and small cohort size, no definite conclusions can be drawn regarding the optimal MAP for ECMO.

*Future research* To fill this knowledge gap RCTs are needed. However, in the short future an RCT will start in the Netherlands, randomizing patients with AMI-CS to either a standard MAP or to a MAP ≥ 55 mmHg. This will be the first step towards determining optimal MAP targets in CS patients. Afterwards, the same study design might be considered to define MAP targets in patients on VA-ECMO.

#### #6: Adjuvant treatments: blood transfusion regimen

Transfusion guidelines for VA-ECMO patients are limited to expert-opinion statements in the ELSO guidelines [1]. Advised thresholds are quite liberal, resulting in a high inter-center variance in thresholds used [41]. Current literature consists mainly of observational studies.

##### Red blood cells (RBC)

RBC transfusion during VA-ECMO is common: 82–100% of patients receive RBC transfusion with a mean of 24 RBC units per run [42–45]. The hemoglobin (Hb) thresholds described are relatively liberal [46]. One of the hypotheses for these liberal thresholds in VA-ECMO is that patients with cardiac failure can develop tissue hypoxemia due to reduced cardiac output. The resulting decreased delivery of oxygen (DO_2_) can be compensated by providing a larger Hb buffer. This, however, does not consider that by the blood flow created by VA-ECMO, a fixed cardiac output, and thus DO_2_, can largely be maintained. Evidence to either confirm or refute this hypothesis is lacking.

##### Platelets

Thrombocytopenia and impaired platelet function are common during VA-ECMO [47, 48]. ELSO guidelines recommend platelet transfusion to maintain a platelet count over 80 × 10^9^/L [1]. Platelet transfusion occurs in 20–50% of patients, while reasons for transfusion are not described [42, 49]. Both severity of thrombocytopenia and platelet transfusion have been associated with mortality [49, 50].

##### Plasma

ELSO guidelines state that indications for plasma transfusion include (i) (suspicion of) decreased antithrombin levels; (ii) correction of coagulation disturbances or (iii) system priming [1]. One-third of patients receive plasma transfusions; however, reasons and indications for transfusion are not properly recorded [42, 51]. As the role of plasma in treating hemorrhage is disputable, while increasing the risk of transfusion-related complications, routine use of plasma should be well considered [52, 53].

##### Hemorrhage

Hemorrhage is one of the main complications during VA-ECMO, occurring in up 60% of patients [54–56]. Central cannulation increases the risk of a hemorrhagic event, with an occurrence rate of 52% vs. 33% in peripheral cannulation [57]. Hemorrhage is associated with all types of transfusion and mortality on ECMO [58]. Therefore, preventing hemorrhage should be a priority in this vulnerable population.

##### Future research

Currently, no prospective studies focusing on transfusion thresholds have been announced. We recommend that reasons for transfusion of RBC, platelets and plasma should be further explored. In other non-ECMO patients, using a restrictive Hb threshold for RBC transfusion has been shown to be safe [59, 60]. As RBC transfusion is most commonly transfused in patients on ECMO, future research should first focus on the optimal Hb threshold for RBC transfusion in VA-ECMO.

#### #7 Adjuvant treatments: anticoagulant therapies

Hemostasis during ECMO is a precarious balance: While (systemic) anticoagulation is needed to prevent thrombotic complications, hemorrhage remains one of the main complications during ECMO. Different therapies, targets and monitoring options can be used, however, this PICO will focus on therapies only.

##### Unfractionated heparin

Unfractionated heparin (UFH) is one of the classic and most commonly used anticoagulant agents during ECMO [61]. However, it comes along with a risk of developing heparin-induced thrombocytopenia (HIT), and recommendations regarding the ideal targets are pending. Several cohort studies have described the safe use of lower UFH anticoagulation targets, showing a decrease of hemorrhagic events without an increase of thrombotic events [62–64].

##### Low-molecular weight heparin

Low-molecular weight heparin (LMWH) has been shown to be safe and effective in renal replacement therapy (RRT) [65]. The past years, a handful of retrospective studies appeared on LMWH in ECMO, showing an equal rate of hemorrhage and reduced thrombotic event rate in favor of LMWH [66]. Prospective studies, however, are lacking.

##### Direct thrombin inhibitors (DTIs)

The use of DTIs in ECMO has been increasing, despite the shortage of prospective studies, mainly indicated in case of suspected HIT. Reports of the use of argatroban in VA-ECMO are limited to case series [67]. More is known about bivalirudin, of which observational retrospective studies show the safe use with regard to hemorrhagic and thrombotic complications [68, 69].

##### Absent systemic anticoagulation

As a result of improved ECMO coating, rationale has shifted towards lower anticoagulation targets. Some have taken it even further, describing the safe withhold of anticoagulation in VA-ECMO [70]. However, limitations include a retrospective design and small sample sizes, thereby limiting generalizability.

##### Future research

One pilot study has been performed comparing UFH targets. Currently, one RCT is ongoing with expected results early 2024, comparing two systemic heparin regimes (high and low target) and LMWH (NCT04536272) [71]. Further work is required to evaluate the best fit anticoagulant therapy and targets in VA-ECMO.

#### #8 Complications: endothelial activation and damage

ECMO induces a systemic inflammatory response due to among others exposure of the patient’s blood to the foreign surface of the ECMO circuit. This results in a variety of coagulative and inflammatory cascades and complex interactions with the endothelium [72].

##### Adhesion molecules and selectins

Activated endothelium is characterized by overexpression of adhesion molecules, such as ICAM1 and VCAM1, and selectins (P-selectin and E-selectin) that facilitate leukocyte adhesion, rolling, and transmigration of activated neutrophils. So far, neither adhesion molecules nor selectins have been studied in adult patients on VA-ECMO.

##### von Willebrand factor

Upon endothelial activation, von Willebrand factor (vWF) is released from the Weibel–Palade bodies [73]. In VA-ECMO patients, vWF antigen levels were high compared to values in healthy controls and remained high within the first 5 days after initiation of ECMO [74].

##### Angiopoietin-2 and VEGF

Also released from Weibel–Palade bodies is angiopoietin-2. Angiopoietin-2 is a growth factor and associated with increased endothelial permeability and organ dysfunction in patients on cardiopulmonary bypass (CPB) [75, 76]. Although angiopoietin-2 levels remained stable within the first three days on VA-ECMO, angiopoietin-2 levels were significantly higher in non-survivors compared to survivors [77]. Within the same study, vascular endothelial growth factor (VEGF), a pro-inflammatory growth factor that enhances endothelial permeability, increased over the first three days of VA-ECMO support. Interestingly, VEGF was lower in non-survivors compared survivors of VA-ECMO support [77].

##### Thrombomodulin

Thrombomodulin is a thrombin receptor on endothelial cells and released after injury [78]. In patients on VA-ECMO, no differences were found in soluble thrombomodulin levels over time nor between survivors and non-survivors [77].

##### Extracellular vesicles

Upon endothelial activation, extracellular vesicles (EVs) are released, which can mediate intercellular communication. Patients on VA-ECMO had increased levels of endothelial-derived EVs after ECMO initiation compared to healthy controls [79]. A follow-up study showed that endothelial-derived EVs did not differ between survivors and non-survivors [80]. Interestingly, EVs derived from leukocytes were associated with outcome [80]. These specific EVs are suggested to induce endothelial dysfunction [81].

##### Future research

Activation of the endothelium seems a less well recognized complication during ECMO, even though it is known from patients on CPB that it is associated with organ dysfunction [76]. The above-described results show preliminary evidence of endothelial activation in patients on VA-ECMO. Although inflammation and endothelial activation might be prices to pay for the benefits of ECMO, we do however recommend further exploration to possibly counteract the detrimental effects. Lastly, in other critically ill patient populations, recently successful evaluation of pharmacological interventions in a translational matter have been performed (i.e., protein kinase inhibitors in acute respiratory distress syndrome). This approach may also be applied to identify and prevent the negative effects of endothelial activation and damage.

## Discussion and conclusion

This scoping review describes a selection of eight high-priority topics in which further research should be performed in patients receiving VA-ECMO, as identified by an expert panel. The expert panel primarily identified almost fifty topics for further elucidation, thereby emphasizing the many current knowledge gaps and research priorities. Available guidelines specific to patients receiving VA-ECMO are scarce, and in the ones available, many topics are based on expert-opinion only. This does not come as a surprise, as for some topics, only one study on the subject could be found. There is a strong need for further evidence-based research in this critically ill patient population.

For all sub-topics, the majority of studies consisted of an observational design. These observational studies often had a retrospective design, and were performed in a single-center setting. This comes along with different disadvantages, including a high risk of different types of bias (i.e., selection, immortal time), confounding and even methodological errors. Moreover, the single-center design impedes translation to other centers or countries, even more when taking into account the lack of evidence-based guidelines. For example, in case of ECPR, due to a different hospital occupancy per country, time-to-ECMO can differ drastically and thereby influence the result either too positive (short time-to-ECMO, high amount of expert centers and resources) or too negative.

Only a handful of RCTs have been performed on patients receiving ECMO. However, those are limited to either respiratory support (i.e., venovenous ECMO) or ECPR. Of these RCTs, stopping early due to meeting the stopping criteria, either for futility or superiority, is not uncommon [12, 13]. RCTs in VA-ECMO can face multiple important obstacles. Firstly, due to the wide range of indications, the patient population receiving VA-ECMO shows a high amount of heterogeneity: for example, ECPR and failure to wean cardiopulmonary bypass may come with different aspects of clinical attention and prognoses. Secondly, multicenter cooperation is key for a feasible study, whereas the number of runs performed by a center can range from a few to high-output. Lastly, ethical considerations play a large role, as patients are per definition unconscious and thus informed consent is dependent on by-proxy or deferred consent strategies.

As a result of the lack of studies in patients receiving VA-ECMO, the next best option consists of the translation and transposition of studies performed in similar patient populations. Although this may be sufficient in some cases, this does not always apply. Different research strategies are required to answer the topics as stated in this review. For example, a translational approach is key in further studying endothelial activation, wherein an essential step is the forming and testing of hypotheses in in vitro and animal models. To study the more general topics, such as cannulation method, it may be sufficient to focus primarily on large observational studies or RCTs as it involves all patients receiving VA-ECMO. Alternative subjects such as choice of anticoagulation on the other hand are preferred to be performed in a homogenous population, whereas a different profile of coagulation disturbances and thus bleeding risk may play an important role.

Currently, several RCTs are ongoing and results are expected within the upcoming years (NCT04620070, NCT04536272). However, with the yearly increasing amount of ECMO centers, indications for VA-ECMO and thus runs and numbers of patients supported with VA-ECMO, more research is needed on a shorter notice. Alternative study designs, such as adaptive platform trials, may be of use in evaluating different adjuvant treatments in VA-ECMO simultaneously and efficiently, such as combining transfusion regimen and evaluating microcirculatory disturbances.

In conclusion, this scoping review identifies and presents an overview of research gaps and priorities in VA-ECMO. Gaining data from translational high-quality research, ranging from preclinical and animal studies to RCTs is important to improve the standard practices in this patient population.

## Supplementary Information

**Additional file 1.** Supplementary materials.

## Data Availability

Not applicable.

## Abbreviations

ACS: Acute coronary syndrome
AMI: Acute myocardial infarction
CPB: Cardiopulmonary bypass
CPR: Cardiopulmonary resuscitation
CS: Cardiogenic shock
DPC: Distal perfusion catheter
DTI: Direct thrombin inhibitor
ECPR: Extracorporeal cardiopulmonary resuscitation
ED: Emergency department
ELSO: Extracorporeal Life Support Organization
Hb: Hemoglobin
HIT: Heparin-induced thrombocytopenia
ICAM-1: Intercellular adhesion molecule-1
INTERMACS: Interagency Registry for Mechanically Assisted Circulatory Support
LMWH: Low-molecular weight heparin
MAP: Mean arterial pressure
MCS: Mechanical circulatory support
NIRS: Near-infrared spectroscopy
PCI: Percutaneous coronary intervention
PICO: Patient/population, intervention, comparison and outcomes
RBC: Red blood cell
RCT: Randomized controlled trial
RRT: Renal replacement therapy
SCAI: Society for Cardiovascular Angiography and Interventions
UFH: Unfractionated heparin
VA-ECMO: Veno-arterial extracorporeal membrane oxygenation
VCAM1: Vascular cell adhesion molecule 1
VEGF: Vascular endothelial growth factor
vWF: von Willebrand factor
