# Supplementary material for: Knowledge gaps and research priorities in adult veno-arterial extracorporeal membrane oxygenation: a scoping review
Source: Intensive Care Med Exp. 2022 Nov 25;10:50. doi: 10.1186/s40635-022-00478-z (PMC9691798; doi:10.1186/s40635-022-00478-z)
Supplement: Supplementary file 1 — Additional file 1. Supplementary materials. [file 40635_2022_478_MOESM1_ESM.docx]

Supplementary materials to

**Knowledge Gaps and Research Priorities in Veno Arterial Extracorporeal Membrane Oxygenation: A Scoping Review**

Senta Jorinde Raasveld, Carolien Volleman, Alain Combes, Lars Mikael Broman, Fabio Silvio Taccone, Elma Peters, Sanne ten Berg, Charissa E. van den Brom, Holger Thiele, Roberto Lorusso, José P.S. Henriques, Alexander P.J. Vlaar

Table of Contents

[Overview expert panel *(alphabetical order)* 2](#_Toc113889707)

[List of topics + PICOs 3](#_Toc113889708)

[Search + flow chart per topic 9](#_Toc113889709)

[Topic 1. Cardiogenic shock: definition and degree of it 9](#_Toc113889710)

[Topic 2. Indication: Selection Criteria for ECPR 13](#_Toc113889711)

[Topic 3. Percutaneous versus surgical cannulation methods 15](#_Toc113889712)

[Topic 4. Monitoring: daily therapy goals 20](#_Toc113889713)

[Topic 5. Monitoring | Optimal balance blood pressure – vasoactive medication, “less is more?” 22](#_Toc113889714)

[Topic 6. Blood transfusion regimen 26](#_Toc113889715)

[Topic 7. Adjuvant treatments: anticoagulant therapy 32](#_Toc113889716)

[Topic 8. Complications: endothelial activation and damage 35](#_Toc113889717)

# Overview expert panel *(alphabetical order)*

| **Name** | **Medical background** | **Department** | **Research lines** |
| --- | --- | --- | --- |
| *Charissa van den Brom* | Medical biology, cardiovascular research | Laboratory of Experimental Intensive Care and Anesthesiology | Tissue perfusion in critically ill patients |
| *Lars Mikael Broman* | Anesthesia, Intensive Care | Extracorporeal Membrane Oxygenation Centre | Clinical and transport aspects of ECMO |
| *Alain Combes* | Cardiology, Intensive Care | Adult Intensive Care | Care of the critically ill cardiac patient  MCS, ECMO  Rescue therapies for severe respiratory failure  Infections in critically ill patients |
| *José P. S. Henriques* | Cardiology | Cardiology | Acute myocardial infarction  Cardiogenic shock  MCS, ECMO |
| *Roberto Lorusso* | Cardiothoracic surgery, intensive care | Heart & Vascular Centre | ECMO: preventing complications, innovation, combining devices, neurologic protection  Cardiac valve replacement  Mechanical complications AMI  Endocarditis  Pacemaker dependency after cardiac surgery  Metabolic syndrome and cardiac surgery |
| *Fabio Silvio Taccone* | Internal Medicine,  Intensive Care | Adult Intensive Care | Antibiotic pharmacokinetics  Brain injury  Microcirculation during severe infections  Therapeutic hypothermia  ECMO |
| *Holger Thiele* | Cardiology | Heart Center: Internal Medicine/Cardiology, Adult Intensive Care | Acute myocardial infarction  Cardiogenic shock  MCS  Acute cardiovascular care |
| *Alexander P.J. Vlaar* | Internal Medicine, Intensive Care | Adult Intensive Care | Transfusion  TRALI/TACO  ECMO  Sepsis  AI Hemodynamics |

# List of topics + PICOs

| **#** | **Topic** | **Population/ problem/ patient** | **Intervention** | **Comparison/ control** | **Outcome** | **Domain** | **Subgroups** |
| --- | --- | --- | --- | --- | --- | --- | --- |
| **1. Indication for ECMO** | | | | | | | |
| 1 | (Massive) pulmonary embolism | (Massive) pulmonary embolism | VA-ECMO | Removal of embolism (see subgroups) | 28-day mortality/ thromboembolic complications/ hemorrhagic complications | Treatment | Surgical thrombectomy Thrombolytic therapy  Other:… |
| 2 | Selection criteria for ECPR | Cardiac arrest | VA-ECMO, selection criteria see subgroups | No VA-ECMO | 28-day mortality/overall mortality/neurologic outcome | Treatment | Age < 65 / > 65  Duration of ongoing resuscitation > 60 / < 60 min On-site/in-hospital insertion Duration of advanced cardiac life support without ROSC  Other:… |
| 3 | Cardiogenic shock: definition and degree of | Cardiogenic shock | VA-ECMO in refractory shock | VA-ECMO in shock | 28 day mortality, shock reversal, ECMO survival, ECMO duration, ICU LOS | Treatment | Definition of use:  INTERMACS  SCAI Shock  Other |
| 4 | Refractory septic shock | Refractory septic shock | VA-ECMO | No VA-ECMO/alternative mechanical support devices | 28 day mortality, shock reversal, ECMO survival, ECMO duration, ICU LOS | Treatment |  |
| 5 | Heart transplantation (pre/post) | Heart transplantation (HTx) | Preventive ECMO initiation/ start ECMO pre-HTx | Start ECMO post-HTx | 28-day mortality, ECMO survival, ECMO duration, successful weaning, ICU LOS | Treatment |  |
| 6 | VAD insertion | Cardiac failure | VA-ECMO as bridge to VAD  OR  VA-ECMO next to VAD | Direct VAD insertion  OR Solely VAD | 28-day mortality, ECMO survival, ECMO duration, successful weaning, ICU LOS | Treatment |  |
| 7 | VA-ECMO in case of insufficient oxygenation by VV-ECMO | Patient on VV-ECMO and remaining hypoxemia | Switch mode to VA-ECMO | Remain VV-ECMO | 28-day mortality, ECMO survival, ECMO duration, successful weaning, ICU LOS | Treatment |  |
| *Ethics* | | | | | | | |
| 8 | Postcardiotomy ECMO: do not mechanically support as addition to DNR/DNI? | Patient undergoing cardiothoracic surgery | "Do mechanically support": VA-ECMO | "Do NOT mechanically support": No VA-ECMO | - | Treatment |  |
| **2. Initiation of ECMO** | | | | | | | |
| *ECMO preparation* | | | | | | | |
| 9 | Priming: type of | Patient undergoing cannulation for VA-ECMO | Crystalloid priming fluid + colloid | Crystalloid priming fluid (0.9% NaCl) | Fluid resuscitation / RBC transfusion / oxygenator lifespan | Prevention |  |
| 10 | Priming: autopriming to facilitate initiation | Patient undergoing (emergent) cannulation for VA-ECMO | Autopriming system | Non-automated priming system | 28-day mortality/oxygenator change/oxygenator lifespan | Prevention |  |
| 11 | Coating: type of | Patient undergoing cannulation for VA-ECMO | Biopassive active coated circuit (see subgroups) | Heparin coated circuit | Clotting time / hemorrhagic/thrombotic complications/oxygenator life span | Prevention | Albumin Polyethylene glycol Phosphorylcholine Poly(2-methoxyethyl acrylate) |
| *ECMO connecting* | | | | | | | |
| 12 | Sweep gas flow: how quickly can you correct? | Initiation of ECMO | Increasing SGF "quickly" (SGF:BF ratio positive) | Standard SGF increase (i.e. 2L SGF and 2L BF/min) | 28-day mortality/neurologic complication rate/ECMO survival/ECMO duration/ICU LOS | Prevention |  |
| 13 | Percutaneous vs surgical cannulation methods | Patient undergoing cannulation for VA-ECMO | Percutaneous cannulation | Surgical cannulation | 28 day survival / ECMO survival/ECMO duration/ICU LOS | Treatment |  |
| 14 | Central vs peripheral cannulation | Patient undergoing cannulation for VA-ECMO | Peripheral cannulation | Central cannulation | 28-day mortality / limb ischemia/ECMO survival/ECMO duration/ICU LOS | Treatment |  |
| 15 | Uni- vs bilateral cannulation | Patient undergoing cannulation for VA-ECMO | Unilateral cannulation | Bilateral cannulation | 28-day mortality / limb ischemia/ ECMO survival/ECMO duration/ICU LOS | Treatment |  |
| 16 | Cannulation strategies: configuration | Patients eligible for VA-ECMO | V-AV as standard ECMO configuration | VA-ECMO configuration | 28-day mortality / limb ischemia/ ECMO survival/ECMO duration/ICU LOS | Treatment |  |
| 17 | Standard distal perfusion cannula in all? | Patient undergoing cannulation for VA-ECMO | Distal perfusion cannula standard | Absence of distal perfusion cannula | 28-day mortality / limb ischemia/ ECMO survival/ECMO duration/ICU LOS | Prevention |  |
| 18 | Prophylactic antibiotics | Patient undergoing cannulation for VA-ECMO | Prophylactic antibiotics for canulation procedure | No prophylactic antibiotics | (Suspected) infection related to cannulation procedure/ sepsis/28-day mortality | Prevention |  |
| **3. ECMO Support care** | | | | | | | |
| *Mechanical support* | | | | | | | |
| 19 | Circulatory: concomitant LV Unloading – in all? | Patients supported with VA-ECMO | Concomitant LV unloading | No concomitant LV unloading | 28-day mortality/efficacy/timing/safety | Treatment | Peripheral ventricular assist device |
| 20 | Venting criteria? | Patients supported with VA-ECMO | Venting (see subgroups) | - | 28-day mortality/efficacy/timing/safety | Treatment | Elevated PCWP Distended, hypo- contractile LV or stagnation of blood in LV as seen on ultrasound AV that remains closed throughout cardiac cycle Marginal oxygenation  Persistent pulmonary edema (X-ray/lung ultrasound) Refractory Ventricular arrhythmias  Other:… |
| 21 | Which venting strategy in which patients? | Patients supported with VA-ECMO | Venting strategy (see subgroups) | No venting strategy | 28-day mortality/efficacy/timing/safety | Treatment | IABP Septostomy/trans-septal balloon/puncture  Tandemheart Impella  Other:… |
| *Monitoring* | | | | | | | |
| 22 | Heart/circulation: standard Swan-Ganz in all patients | Patient supported with VA-ECMO | Swan Ganz as standard part of HD monitoring | No Swan Ganz as part of HD monitoring | 28 day mortality, shock reversal, ECMO run duration, ICU LOS, ECMO survival | Treatment |  |
| 23 | Heart/circulation: standard daily ultrasound | Patient supported with VA-ECMO | Standard daily cardiac ultrasound | Infrequent/non-standard cardiac ultrasound | 28-day mortality/cardiac function/ cardiac recovery/ ECMO run duration | Treatment |  |
| 24 | Heart/circulation: therapy goals | Patient supported with VA-ECMO | Daily goals | Standard care | 28 day mortality, ECMO survival/ECMO duration/ICU LOS | Treatment | Min/max fluid balance  Reducing blood flow  Reducing sedatives  Others:… |
| 25 | Heart/circulation: hemodynamic measures | Patient supported with VA-ECMO | Advanced monitoring | Standard care | 28 day mortality, ECMO survival/ECMO duration/ICU LOS | Treatment | Swan Ganz  Flotrac ®  Others: … |
| 26 | Heart/circulation: biochemical measures | Patient supported with VA-ECMO | Lactate driven therapy | Standard care | 28 day mortality, shock reversal, ECMO run duration, ICU LOS | Treatment |  |
| 27 | Heart/circulation: optimal balance blood pressure – vasoactive medication (“less is more?”) | Patient supported with VA-ECMO | Lower MAP | MAP 65 mmHg | 28 day mortality, shock reversal, ECMO run duration, ICU LOS | Treatment |  |
| 28 | Neuro: Neuromonitoring | Patient supported with VA-ECMO | Neuromonitoring (see subgroups) | No neuromonitoring | 28 day mortality, ECMO survival/ECMO duration/ICU LOS/neurological complications | Treatment | EEG: intermittent  EEG: continuous  NIRS Cranial ultrasound Transcranial Doppler  Scheduled standard CAT/MRI scan  Other:… |
| 29 | Leg ischemia | Patient supported with VA-ECMO | Monitoring (see subgroups) | No monitoring | Leg ischemia / leg amputation/28 day mortality, ECMO survival/ECMO duration/ICU LOS | Treatment | NIRS  Doppler ultrasonography (intermittent)  Ultrasound (intermittent) Flow measurement at  distal canula  Other:… |
| *Adjuvant treatments* | | | | | | | |
| 30 | Monitoring anticoagulant therapy (i.e. viscoelastic testing) | Patient supported with VA-ECMO | Anticoagulant monitoring (see subgroups) |  | Hemorrhagic/thrombotic complications/28 day mortality, ECMO survival/ECMO duration/ICU LOS | Treatment | Standard lab testing (aPTT, PT, ACT) Viscoelastic testing: ROTEM Viscoelastig testing: TEG Coagulation testing: fibrinogen levels, specific coagulation factors  Other:… |
| 31 | Blood transfusion regimen (RBC, platelets) | Patient supported with VA-ECMO | Restrictive (threshold dependent on blood type) | Liberal (threshold dependent on blood type) |  | Treatment | Red blood cells Platelets Plasma  Other:… |
| 32 | Anticoagulation type | Patient supported with VA-ECMO | Heparin intravenous continuously | Other: see subgroups | 28-day mortality/ thromboembolic complications/ hemorrhagic complications | Treatment | LMWH DTIs  Absent |
| 33 | Anticoagulation limits | Patient supported with VA-ECMO | Heparin intravenous continuously | Other: see subgroups | 28-day mortality/ thromboembolic complications/ hemorrhagic complications | Treatment | LMWH DTIs  Absent |
| 34 | Renal replacement therapy: parallel/in series connection RRT | Patient supported with VA-ECMO and indication for renal replacement therapy | Separate iv access for RRT and ECMO ("parallel") | Direct connection between RRT and ECMO ("in-series") | Duration of support /28 day mortality, ECMO survival/ECMO duration/ICU LOS | Treatment |  |
| 35 | Inotropes, vasopressors and pulmonary vasoregulation | Patient on VV and VA-ECMO | V receptor vasopressor | Alfa receptor vasopressor | Shock reversal/28 day mortality, ECMO survival/ECMO duration/ICU LOS | Treatment | Right ventricular function TAPSE |
| 36 | Therapeutic Drug Monitoring (e.g. antibiotics); clearance by ECMO filter | Patients supported with VA-ECMO | Additional measurements of antibiotics | Standard measurements | Infection rate/ toxic levels medication/ low levels medication/28 day mortality, ECMO survival/ECMO duration/ICU LOS | Treatment |  |
| *Complications* | | | | | | | |
| 37 | Systemic inflammation | Patients supported with VA-ECMO | Measuring TNF, IL-6 to monitor systemic inflammation | No additional measurements | 28 day mortality, ECMO survival/ECMO duration/ICU LOS | Diagnostics | TNF IL 6 |
| 38 | Endothelial activation and damage | Patients supported with VA-ECMO | Measuring ICAM-1, VCAM, Syndecan to monitor endothelial activation and damage | No additional measurements | 28 day mortality, ECMO survival/ECMO duration/ICU LOS | Diagnostics | ICAM-1, VCAM, Syndecan |
| 39 | Microcirculatory perfusion disturbances and tissue oxygenation | Patients supported with VA-ECMO | Standard ICDF Camera area measurements | No standard additional measurements | 28 day mortality, ECMO survival/ECMO duration/ICU LOS | Diagnostics | ICDF camera area measurements |
| *Multidisciplinary approach* | | | | | | | |
| 40 | Nursing aspects | Patients supported with VA-ECMO | 1:1 nursing | 1:2 nursing | 28-day mortality / length of hospital stay/ECMO survival/ ICU LOS | Treatment |  |
| 41 | Active physical therapy during support | Patients supported with VA-ECMO | No physical therapy while on ECMO | Active mobilization while on ECMO | 28-day mortality / length of hospital stay/ECMO survival/ ICU LOS | Treatment |  |
| 42 | Feeding | Patients supported with VA-ECMO | Enteral feeding | Parenteral feeding | 28-day mortality / length of hospital stay/ECMO survival/ ICU LOS | Treatment | Early enteral feeding |
| **4. End of ECMO support** | | | | | | | |
| *Weaning* | | | | | | | |
| 43 | Drugs to facilitate weaning | Patients on VA-ECMO eligible for weaning | Inotropic drugs continued | No inotropes | 28-day mortality / length of hospital stay/ECMO survival/ ICU LOS/successful weaning | Treatment |  |
| 44 | Weaning criteria | Patients on VA-ECMO eligible for weaning | Unsuccessful weaning from VA-ECMO | Successful weaning from VA-ECMO | Successful weaning/28-day mortality / length of hospital stay/ECMO survival/ ICU LOS | Treatment | Echocardiography: improved RVF and LVF  Absent or low amount of inotropic support Extubated yes/no Additional MCS weaned yes/no  Other:… |
| 45 | Monitoring successful weaning | Patients on VA-ECMO eligible for weaning | Advanced monitoring | standard care | Successful weaning/28-day mortality / length of hospital stay/ECMO survival/ ICU LOS | Treatment | CVP, VTI, Wedgepressure, cardiac ultrasound TAPSE  Other:… |
| *Decannulation* | | | | | | | |
| 47 | Surgical vs percutaneous removal | Patients weaned from VA-ECMO | Percutaneous decannulation | Surgical decannulation | 28-day mortality / hemorrhage at cannula site /  cannula site infection | Prevention |  |
| 48 | Optimal percutaneous techniques | Patients weaned from VA-ECMO | MANTA plug-based vascular closure device | Perclose ProGlide closure device | Hemorrhage at cannula site/ conversion to surgical decannulation | Treatment |  |
| *Outcomes* | | | | | | | |
| 49 | Prognostic survival models | Patients supported with VA-ECMO | Survival Score | // | Prognostic accuracy | Prognosis |  |

# Search + flow chart per topic

## Topic 1. Cardiogenic shock: definition and degree of it

**Indication | Cardiogenic shock: definition and degree of it**

Mesh terms

- Shock, Cardiogenic
  - Shock, Cardiogenic/mortality
  - Shock, Cardiogenic/therapy
- Extracorporeal membrane oxygenation
  - Extracorporeal membrane oxygenation/mortality
  - Extracorporeal membrane oxygenation/therapy

Search: PubMed on 02-08-2022

#1
( "Shock, Cardiogenic/mortality"[Mesh] OR "Shock, Cardiogenic/therapy"[Mesh] OR “Cardiogenic shock” [tiab] OR “INTERMACS” [tiab])

**AND**

#2
( "Extracorporeal Membrane Oxygenation/mortality"[Mesh] OR "Extracorporeal Membrane Oxygenation/therapeutic use"[Mesh] OR "Extracorporeal Membrane Oxygenation/therapy"[Mesh] OR “Venoarterial ECMO” [tiab] OR “VA ECMO” [tiab] OR “ECPR” [tiab])

**NOT**

#3
(“Pediatrics” [Mesh] OR “pediatric*” [tiab] OR “paediatric*” [tiab] OR “children*”[tiab] OR “neonatal” [tiab] OR “NICU” [tiab])

**AND**

#4
(“Timing” [tiab] OR “early” [tiab] OR “late” [tiab] OR “refractory shock”[tiab] OR “shock reversal” [tiab] OR “Mechanical support” [tiab] OR “mechanical cardiac support” [tiab])

**AND**

#5 ("myocardial infarction"[MeSH Terms] OR ("myocardial"[All Fields] AND "infarction"[All Fields]) OR "myocardial infarction"[All Fields])

Results Pubmed
*#1 and #2 results in 956 hits
#1, #2 and #3 results in 904 hits
#1, #2, #3 and #5 results in 159 hits
#1, #2 and #4 results in 225 hits
#1, #2, #3 and #4 results in 209 hits
#1, #2, #3, #4 and #5 results in 153 hits*


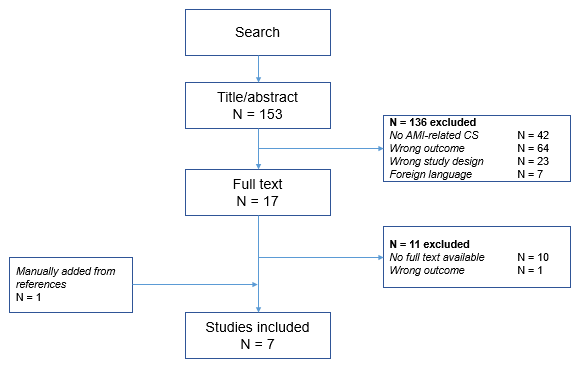


| **Title** | **Year** | **Journal** | **Authors** | **doi** |
| --- | --- | --- | --- | --- |
| Association Between Timing of Extracorporeal Membrane Oxygenation and Clinical Outcomes in Refractory Cardiogenic Shock. | 2021 | JACC. Cardiovascular interventions | Lee HH, Kim HC, Ahn CM, Lee SJ, Hong SJ, Yang JH, Kim JS, Kim BK, Ko YG, Choi D, Gwon HC, Hong MK, Jang Y | - [10.1016/j.jcin.2021.03.048](https://doi.org/10.1016/j.jcin.2021.03.048) |
| Optimal Timing of Venoarterial-Extracorporeal Membrane Oxygenation in Acute Myocardial Infarction Patients Suffering From Refractory Cardiogenic Shock. | 2020 | Circulation journal | Choi KH, Yang JH, Hong D, Park TK, Lee JM, Song YB, Hahn JY, Choi SH, Choi JH, Chung SR, Cho YH, Jeong DS, Sung K, Kim WS, Lee YT, Gwon HC | - [10.1253/circj.CJ-20-0259](https://doi.org/10.1253/circj.cj-20-0259) |
| Early predictors of mortality in refractory cardiogenic shock following acute coronary syndrome treated with extracorporeal membrane oxygenator. | 2021 | Journal of artificial organs | Torre T, Toto F, Klersy C, Theologou T, Casso G, Gallo M, Surace GG, Franciosi G, Demertzis S, Ferrari E | - [10.1007/s10047-021-01252-w](https://doi.org/10.1007/s10047-021-01252-w) |
| Association between delay to coronary reperfusion and outcome in patients with acute coronary syndrome undergoing extracorporeal cardiopulmonary resuscitation. | 2017 | Resuscitation | Kuroki N, Abe D, Iwama T , Suzuki K, Sugiyama K, Akashi A, Hamabe Y, Aonuma K, Sato A | - [10.1016/j.resuscitation.2017.02.007](https://doi.org/10.1016/j.resuscitation.2017.02.007) |
| Efficacy of mechanical circulatory support used before versus after primary percutaneous coronary intervention in patients with cardiogenic shock from ST-elevation myocardial infarction: A systematic review and meta-analysis. | 2022 | Cardiovascular revascularization medicine | Del Rio-Pertuz G, Benjanuwattra J, Juarez M, Mekraksakit P, Argueta-Sosa E, Ansari MM | - [10.1016/j.carrev.2022.05.002](https://doi.org/10.1016/j.carrev.2022.05.002) |
| Predictors of clinical outcome after early veno-arterial extracorporeal membrane oxygenation in cardiogenic shock complicating ST-elevation myocardial infarction. | 2021 | Journal of invasive cardiology | Lukasz Szczanowicz L, Majunke N, de Waha-Thiele S, Tietz F, Schürer S, Kirsch K, Desch S, Thiele H, Sandri M |  |
| Optimal timing of extracorporeal membrane oxygenation in patients with acute myocardial infarction complicated by profound cardiogenic shock after resuscitated cardiac arrest | 2018 | European Heart Journal | M.C. Kim, Y.K. Ahn, J.Y. Cho, K.H. Lee, D.S. Sim, H.J. Yoon, N.S. Yoon, K.H. Kim, Y.J. Hong, H.W. Park, J.H. Kim, M.H. Jeong, J.G. Cho, J.C. Park | <https://doi.org/10.1093/eurheartj/ehy564.469> |
| Implementation of extracorporeal membrane oxygenation before primary percutaneous coronary intervention may improve the survival of patients with ST-segment elevation myocardial infarction and refractory cardiogenic shock | 2018 | International journal of cardiology | Huang CC, Hsu JC, Wu YW, Ke SR, Huang JH, Chiu KM, Liao PC | - [10.1016/j.ijcard.2018.07.023](https://doi.org/10.1016/j.ijcard.2018.07.023) |

## Topic 2. Indication: Selection Criteria for ECPR

**Indication | Selection criteria for ECPR**

P – Cardiac arrest

I – VA ECMO, selection criteria (*see subgroups*)

C – No VA ECMO

O – in-hospital mortality, LOS

*With special focus on the following sub-topics*

- Age criterion: cut-off < 65 years
- Duration of ongoing resuscitation without ROSC: cut-off <60 minutes
- Location of insertion: in-hospital or on-site

Mesh-terms

- Extracorporeal membrane oxygenation
- Mesh-terms
  - Extracorporeal membrane oxygenation/mortality
  - Extracorporeal membrane oxygenation/therapy
- Cardiopulmonary resuscitation
  - Cardiopulmonary resuscitation/therapy
  - Cardiopulmonary resuscitation/methods*
  - Cardiopulmonary resuscitation/mortality
  - Cardiopulmonary resuscitation/standards
- Out-of-Hospital cardiac arrest
  - Out-of-Hospital cardiac arrest/mortality
  - Out-of-Hospital cardiac arrest/therapy

Search: PubMed (01-09-2022)

#1
("Out-of-Hospital Cardiac Arrest/mortality"[Mesh] OR "Out-of-Hospital Cardiac Arrest/therapy"[Mesh] OR “OHCA” [tiab] or “out-of-hospital cardiac arrest” [tiab] OR acute myocardia* infarction [tiab])

**AND**

#2

( "Cardiopulmonary Resuscitation/mortality"[Mesh] OR "Cardiopulmonary Resuscitation/standards"[Mesh] OR "Cardiopulmonary Resuscitation/methods"[Mesh] OR "Cardiopulmonary Resuscitation/therapy"[Mesh] OR “extracorporeal cardiopulmonary resuscitation”[tiab] OR “ECPR” [tiab])

**NOT**

#3
(“Pediatrics” [Mesh] OR “pediatric*” [tiab] OR “paediatric*” [tiab] OR “children*”[tiab] OR “neonatal” [tiab] OR “NICU” [tiab])

**AND**

#4
(“Criteria” [tiab] OR “age limit” [tiab] OR “age”[tiab] OR “ROSC” [tiab] OR “return of spontaneous circulation” [tiab] OR “time limit” [tiab] OR “in-hospital” [tiab] OR “on-site” [tiab])

**AND**

#5

(“extracorporeal cardiopulmonary resuscitation”[tiab] OR “ECPR” [tiab])

**AND**

#6

("Cardiopulmonary Resuscitation/mortality"[Mesh] OR "Cardiopulmonary Resuscitation/standards"[Mesh] OR "Cardiopulmonary Resuscitation/methods"[Mesh] OR "Cardiopulmonary Resuscitation/therapy"[Mesh])

Results Pubmed

*#1 and #2 results in 2495 hits
#1, #2 and #3 results in 979 hits
#1, #2 and #4 results in 1198 hits
#1, #2, #3 and #4 results in 1106 hits
After deciding to divide search #2 in two separate parts (#5 and #6).*

*#1, #3, #4 and #5 results in 203 hits*

*#1, #3, #5 and #6 results in 123 hits*

*#1, #3, #4, #5 and #6 results in 82 hits*


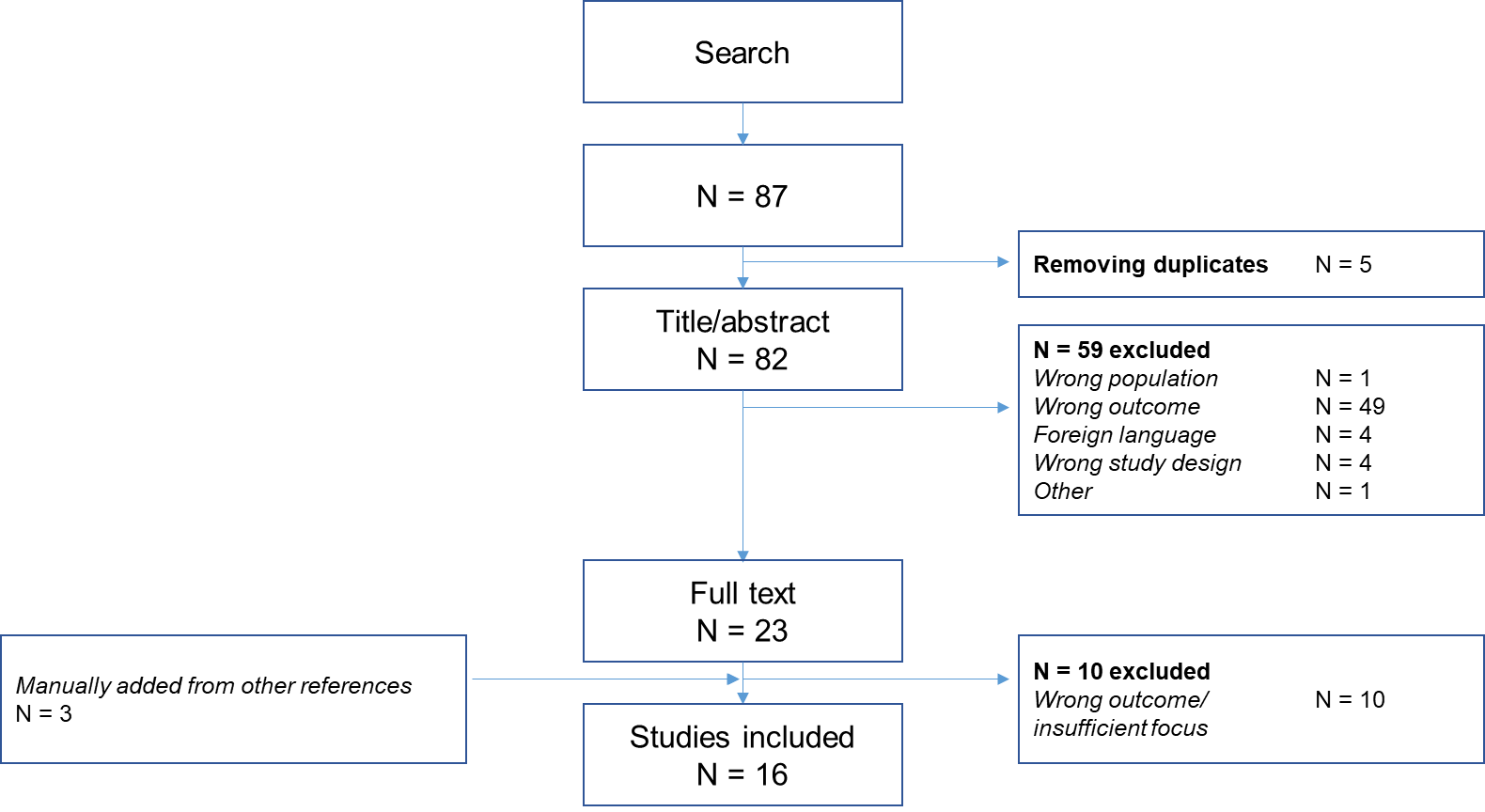


| **Title** | **Year** | **Journal** | **Authors** | **doi** |
| --- | --- | --- | --- | --- |
| Effect of Intra-arrest Transport, Extracorporeal Cardiopulmonary Resuscitation, and Immediate Invasive Assessment and Treatment on Functional Neurologic Outcome in Refractory Out-of-Hospital Cardiac Arrest: A Randomized Clinical Trial | 2022 | JAMA | Belohlavek, Smalcova, Franek et al. | <https://doi.org/10.1001/jama.2022.1025> |
| Advanced reperfusion strategies for patients with out-of-hospital cardiac arrest and refractory ventricular fibrillation (ARREST): a phase 2, single centre, open-label, randomised controlled trial | 2020 | the Lancet | Yannopoulos, Bartos, Raveendran et al. | <https://doi.org/10.1016/S0140-6736(20)32338-2> |
| Descriptive analysis of extracorporeal cardiopulmonary resuscitation following out-of-hospital cardiac arrest-An ELSO registry study. | 2017 | Resuscitation | Haas, Coute, Hsu et al. | <https://doi.org/10.1016/j.resuscitation.2017.08.003> |
| An observational study of extracorporeal CPR for in-hospital cardiac arrest secondary to myocardial infarction | 2014 | Emergency Medicine Journal | Chou, Fang, Yen et al. | <https://doi.org/10.1136/emermed-2012-202173> |
| Early initiation of extracorporeal life support in refractory out-of-hospital cardiac arrest: Design and rationale of the INCEPTION trial. | 2019 | American Heart Journal | Bol, Suverei, Lorusso et al. | <https://doi.org/10.1016/j.ahj.2018.12.008> |
| How effective is extracorporeal cardiopulmonary resuscitation (ECPR) for out-of-hospital cardiac arrest? A systematic review and meta-analysis. | 2022 | The American Journal of Emergency Medicine | Downing, Al Falasi, Cardona et al. | <https://doi.org/10.1016/j.ajem.2021.08.072> |
| Impact of age on the outcomes of extracorporeal cardiopulmonary resuscitation: analysis using inverse probability of treatment weighting. | 2021 | European Journal of Cardio-Thoracic Surgery | Kim, Cho, Yang et al. | <https://doi.org/10.1093/ejcts/ezab339> |
| Impact of extracorporeal cardiopulmonary resuscitation on outcomes of elderly patients who had out-of-hospital cardiac arrests: a single-centre retrospective analysis. | 2018 | BMJ Open | Goto, Morita, Kitamura et al. | <https://doi.org/10.1136/bmjopen-2017-019811> |
| Improved outcome of extracorporeal cardiopulmonary resuscitation for out-of-hospital cardiac arrest--a comparison with that for extracorporeal rescue for in-hospital cardiac arrest. | 2014 | Resuscitation | Wang, Chou, Becker et al. | <https://doi.org/10.1016/j.resuscitation.2014.06.022> |
| Predictors of survival following extracorporeal cardiopulmonary resuscitation in patients with acute myocardial infarction-complicated refractory cardiac arrest in the emergency department: a retrospective study. | 2015 | Journal of Cardiothoracic Surgery | Han, Kim, Choi et al. | <https://doi.org/10.1186/s13019-015-0212-2> |
| Prevalence, natural history, and time-dependent outcomes of a multi-center North American cohort of out-of-hospital cardiac arrest extracorporeal CPR candidates. | 2017 | Resuscitation | Reynolds, Grunau, Elmer et al. | <https://doi.org/10.1016/j.resuscitation.2017.05.024> |
| The association between time to extracorporeal cardiopulmonary resuscitation and outcome in patients with out-of-hospital cardiac arrest. | 2022 | European Heart Journal | Kawakami, Tahara, Koga et al. | <https://doi.org/10.1093/ehjacc/zuac010> |
| Time from arrest to extracorporeal cardiopulmonary resuscitation and survival after out-of-hospital cardiac arrest. | 2019 | Emergency Medicine Australia | Park, Song, Shin et al. | <https://doi.org/10.1111/1742-6723.13326> |
| Extracorporeal membrane oxygenation for refractory cardiac arrest: a retrospective multicenter study. | 2020 | Intensive Care Medicine | Lunz, Calabro, Belliato et al. | <https://doi.org/10.1007/s00134-020-05926-6> |
| Prognostic factors for extracorporeal cardiopulmonary resuscitation recipients following out-of-hospital refractory cardiac arrest. A systematic review and meta-analysis | 2017 | Resuscitation | Debaty, Babaz, Durand et al. | <https://doi.org/10.1016/j.resuscitation.2016.12.011> |
| Out-of-hospital cardiac arrest patients with an initial non-shockable rhythm could be candidates for extracorporeal cardiopulmonary resuscitation: a retrospective study. | 2020 | Scand J Trauma Resusc Emerg Med | Tanimoto, Sugiyama, Tanabe et al. | <https://doi.org/10.1186%2Fs13049-020-00800-2> |

## Topic 3. Percutaneous versus surgical cannulation methods

**ECMO Connecting | Percutaneous vs surgical cannulation methods**

P – Patients undergoing cannulation for VA ECMO

I – Percutaneous cannulation
C – Surgical cannulation

O – Hemorrhage, in-hospital mortality

**Search: PubMed**

Mesh terms

- Extracorporeal membrane oxygenation
- Hemorrhage
  - Hemorrhage/complications
  - Hemorrhage/mortality
  - Hemorrhage/secondary

#1
("Extracorporeal Membrane Oxygenation/mortality"[Mesh] OR "Extracorporeal Membrane Oxygenation/therapeutic use"[Mesh] OR "Extracorporeal Membrane Oxygenation/therapy"[Mesh] OR “Venoarterial ECMO” [tiab] OR “VA ECMO” [tiab] OR “ECPR” [tiab])

**AND**

#2
(“Percutaneous cannulation” [tiab] OR “percutaneous” [tiab] OR “percut*” [tiab] OR “surgical cannulation”[tiab] OR “surgical”[tiab])

**AND**

#3

("Hemorrhage/complications"[Mesh] OR "Hemorrhage/mortality"[Mesh] OR “Hemorrhage/secondary"[Mesh] OR “hemorrhage” [tiab] OR “hemorrhagic” [tiab] OR “haemorrhage”[tiab] OR “haemorrhagic”[tiab] OR “bleeding” [tiab] OR “blood loss” [tiab] OR “bleed*”[tiab])

**NOT**

#4
(“Pediatrics” [Mesh] OR “pediatric*” [tiab] OR “paediatric*” [tiab] OR “children*”[tiab] OR “neonatal” [tiab] OR “NICU” [tiab])

Results
*#1 and #2 results in 715 hits
#1 and #2 and #3 results in 207 hits
#1 and #2 and #3 and #4 results in 150 hits*

**Search: EMBASE**

#1
‘extracorporeal membrane oxygenation’:ti,ab OR ‘ecmo’:ti,ab OR ‘veno-arterial extracorporeal membrane oxygenation’:ti,ab OR ‘va-ecmo’:ti,ab OR ‘va ecmo’:ti,ab OR ‘ecpr’:ti,ab

**AND**

#2
‘percutaneous cannulation’:ti,ab OR ‘percutaneous’:ti,ab OR ‘surgical cannulation’:ti,ab OR ‘surgical’:ti,ab

**AND**

#3
‘hemorrhage’:ti,ab OR ‘hemorhagic’:ti,ab OR ‘haemorrhage’:ti,ab OR ‘haemorrhagic’:ti,ab OR ‘bleeding’:ti,ab OR ‘blood loss’:ti,ab OR ‘bleed’:ti,ab

**NOT**

#4
‘pediatric’:ti,ab OR ‘paediatric’:ti,ab OR ‘children’:ti,ab OR ‘neonatal’:ti,ab OR ‘nicu’:ti,ab

Results Embase

*#1 and #2 results in 4605 hits
#1 and #2 and #3 results in 1060 hits
#1 and #2 and #3 and #4 results in 814 hits*

**Search: Web of Science**

#1

(((((((((((TI=(extracorporeal membrane oxygenation)) OR AB=(extracorporeal membrane oxygenation)) OR TI=(ecmo)) OR AB=(ecmo)) OR TI=(veno-arterial extracorporeal membrane oxygenation)) OR AB=(veno-arterial extracorporeal membrane oxygenation)) OR TI=(va-ecmo)) OR AB=(va-ecmo)) OR TI=(va ecmo)) OR AB=(va ecmo)) OR TI=(ecpr)) OR AB=(ecpr)

**AND**

#2
(((((((TI=(percutaneous cannulation)) OR AB=(percutaneous cannulation)) OR TI=(percutaneous)) OR AB=(percutaneous)) OR TI=(surgical cannulation)) OR AB=(surgical cannulation)) OR TI=(surgical)) OR AB=(surgical)

**AND**

#3
(((((((((((((TI=(hemorrhage)) OR AB=(hemorrhage)) OR TI=(hemorhagic)) OR AB=(hemorhagic)) OR TI=(haemorrhage)) OR AB=(haemorrhage)) OR TI=(haemorrhagic)) OR AB=(haemorrhagic)) OR TI=(bleeding)) OR AB=(bleeding)) OR TI=(blood loss)) OR AB=(blood loss)) OR TI=(bleed)) OR AB=(bleed)

**NOT**

#4
(((((((((TI=(pediatric)) OR AB=(pediatric)) OR TI=(paediatric)) OR AB=(paediatric)) OR TI=(children)) OR AB=(children)) OR TI=(neonatal)) OR AB=(neonatal)) OR TI=(nicu)) OR AB=(nicu)

Results Web of Science
*#1 and #2 results in 2023 hits
#1 and #2 and #3 results in 384 hits
#1 and #2 and #3 and #4 results in 300 hits*


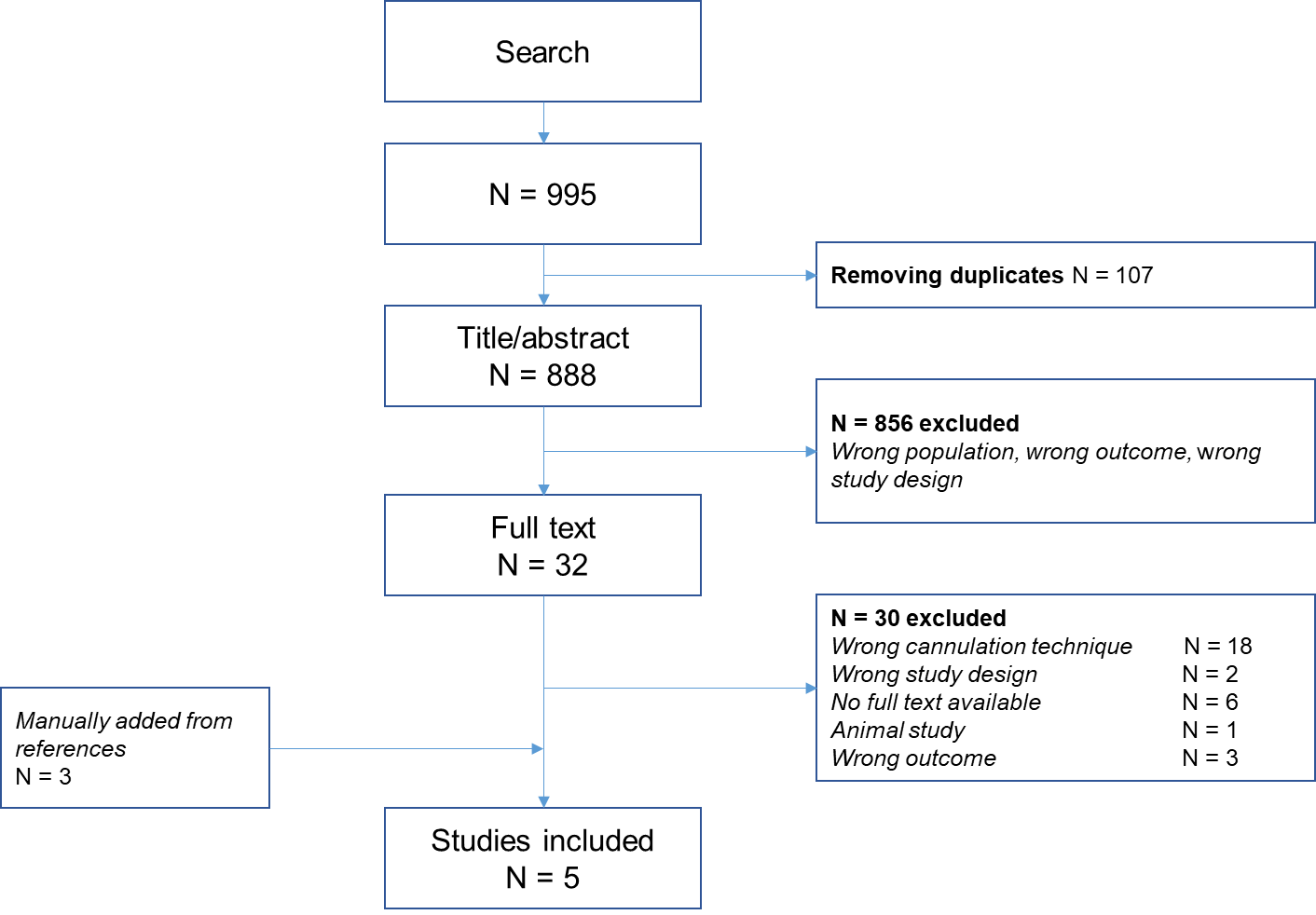


| **Title** | **Year** | **Journal** | **Authors** | **doi** |
| --- | --- | --- | --- | --- |
| Percutaneous versus surgical cannulation for femoro-femoral VA-ECMO in patients with cardiogenic shock: Results from the Extracorporeal Life Support Organization Registry | 2022 | J Heart Lung Transplant | Wang L, Yang F, Zhang S, Li C, Du Z, Rycus P, Tonna JE, Alexander P, Lorusso R, Fan E, Ogino M, Brodie D, Combes A, Chen YS, Qiu H, Peng Z, Fraser JF, Shao J, Jia M, Wang H, Hou X | <https://doi.org/10.1016/j.healun.2022.01.009> |
| Limb ischemia in peripheral veno-arterial  extracorporeal membrane oxygenation: a  narrative review of incidence, prevention,  monitoring, and treatment | 2019 | Critical Care | Bonicolini, Martucci, Simons, Raffa, Spina, Lo Coco, Arcadipane, Pilato, Lorusso | <https://doi.org/10.1186/s13054-019-2541-3> |
| Percutaneous versus surgical femoro-femoral veno-arterial ECMO: a propensity score matched study | 2018 | Intensive Care Med | Danial P, Hajage D, Nguyen LS, Mastroianni C, Demondion P, Schmidt M, Bouglé A, Amour J, Leprince P, Combes A, Lebreton G | <https://doi.org/10.1007/s00134-018-5442-z> |
| Peripheral versus central extracorporeal membrane oxygenation for postcardiotomy shock: Multicenter registry, systematic review, and meta-analysis | 2019 | Journal of Thoracic Cardiovascular Surgery | Mariscalco, Salsano, Fiore, Dalén, Ruggieri, Saeed, Jónsson, Gatti, Zipfel, DellAquila, Perrotti, Loforte, Livi, Pol, Spadaccio, Pettinari, Ragnarsson, Alkahmees, El-Dean, Bounader, Biancari | <https://doi.org/10.1016/j.jtcvs.2019.10.078> |
| Venoarterial Extracorporeal Membrane  Oxygenation for Postcardiotomy  Shock—Analysis of the Extracorporeal Life  Support Organization Registry* | 2021 | Critical Care Medicine | Kowalewski, Zielinski, Brodie, MacLaren, Whitman, Raffa, Boeken, Shekar, Chen, Bermudez, D’Alessandro, Hou, Haft, Belohlavek, Dziembowska, Suwalski, Alexander, Barbaro, Gaudino, Di Mauro, Maessen, Lorusso | DOI: 10.1097/CCM.0000000000004922 |

## Topic 4. Monitoring: daily therapy goals

**Monitoring | Daily therapy goals**

P – Patients supported with VA ECMO

I – Daily goals (*see subgroups*)

C – Standard care

O – In-hospital mortality, LOS

*With special focus on the following sub-topics*

- Min/max fluid balance
- Reducing blood flow
- Reducing sedatives

Mesh-terms

- Extracorporeal membrane oxygenation
  - Extracorporeal membrane oxygenation/mortality
  - Extracorporeal membrane oxygenation/therapy
- Standard of care
- Patient care planning
  - Patient Care Planning/methods
  - Patient Care Planning/standards
- Hypnotics and Sedatives
  - Hypnotics and Sedatives/administration and dosage
  - Hypnotics and Sedatives/standards
  - Hypnotics and Sedatives/therapy

Search: PubMed (10-9-2022)

#1

( "Extracorporeal Membrane Oxygenation/mortality"[Mesh] OR "Extracorporeal Membrane Oxygenation/therapeutic use"[Mesh] OR "Extracorporeal Membrane Oxygenation/therapy"[Mesh] OR “Venoarterial ECMO” [tiab] OR “VA ECMO” [tiab] OR “ECPR” [tiab])

**AND**

#2
(“Standard of care” [Mesh] OR “standard practice” [tiab] OR “standard care” [tiab] OR “standard protocol” [tiab] OR “protocol” [tiab] OR “standard management” [tiab] OR “daily management” [tiab] OR “patient care planning” [Mesh] OR "Patient Care Planning/methods"[Mesh] OR "Patient Care Planning/standards"[Mesh])

**NOT**

#3
(“Pediatrics” [Mesh] OR “pediatric*” [tiab] OR “paediatric*” [tiab] OR “children*”[tiab] OR “neonatal” [tiab] OR “NICU” [tiab])

**AND**

#4
(“Hypnotics and Sedatives”[Mesh] OR "Hypnotics and Sedatives/administration and dosage"[Mesh] OR "Hypnotics and Sedatives/standards"[Mesh] OR "Hypnotics and Sedatives/therapeutic use"[Mesh] OR "Hypnotics and Sedatives/therapy"[Mesh] OR “sedatives” [tiab] OR “sedation”[tiab] OR “propofol” [tiab] OR “dexmedetomidine” [tiab] OR “benzodiazepines” [tiab])

**OR**

#5
(“Fluid balance” [tiab] OR “fluid overload” [tiab] OR “hypervolemia” [tiab] OR “hypervolaemia”[tiab] OR “cumulative fluid” [tiab])

**OR**

#6
(“blood circulation” [Mesh] OR “blood flow” [tiab] OR “reduc* blood flow”[tiab] OR “ECMO flow” [tiab])

Results

*#1 and #2 results in 196 hits
#1, #2 and #3 results in 146 hits
#1, #2 and #4 results in 3 hits
#1, #2, #3 and #4 results in 1 hits*

*#1, #3 and #4 result in 0 hits*

*#1, #3 and #5 result in 26 hits*

*#1, #3 and #6 result in 291 hits*


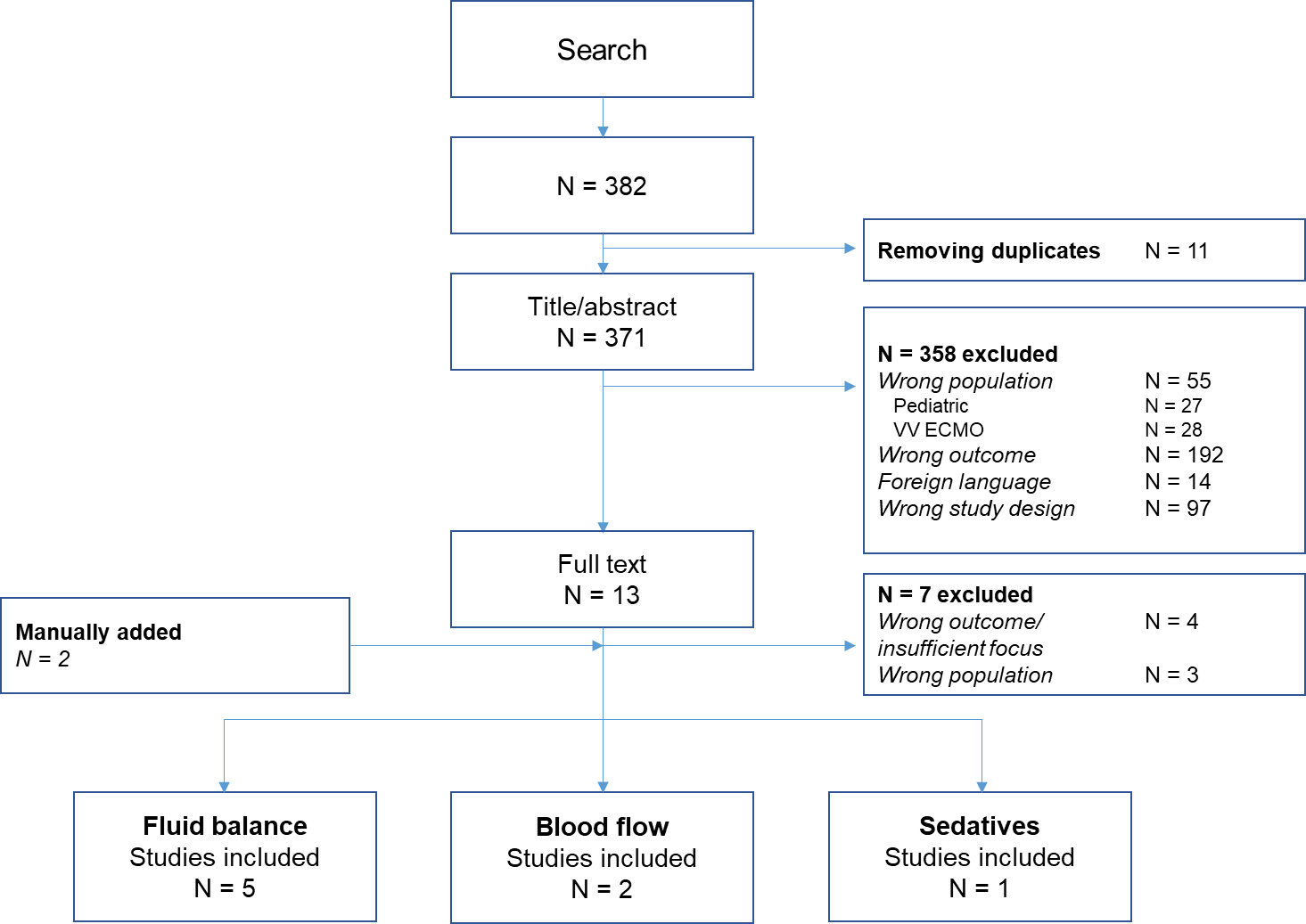


| **Title** | **Year** | **Journal** | **Authors** | **doi** |
| --- | --- | --- | --- | --- |
| Combination of extracorporeal membrane  oxygenation and continuous renal replacement  therapy in critically ill patients: a systematic review | 2014 | Critical Care | Chen, Yu, Yin, Zhou | <https://doi.org/10.1186/s13054-014-0675-x> |
| Fluid Overload and Mortality in Patients with Severe Acute Kidney Injury and Extracorporeal Membrane Oxygenation | 2020 | Kidney360 | Gunning, Kutuby, Rose et al. | <https://doi.org/10.34067/KID.0000402019> |
| Acute Kidney Injury During Extracorporeal Membrane Oxygenation: Timing and Fluid Overload May Be Important Determinants of Outcome* | 2015 | Critical Care Medicine | Cerdá | DOI: 10.1097/CCM.0000000000001172 |
| Extracorporeal Life Support Organization Guidelines for Fluid Overload, Acute Kidney Injury, and Electrolyte Management | 2022 | ASAIO Journal | Bridges, Dhar, Ramanathan et al. | DOI: 10.1097/MAT.0000000000001702 |
| Impact of fluid balance on outcome of adult  patients treated with extracorporeal membrane  oxygenation | 2014 | Intensive Care Medicine | Schmidt, Baily, Kelly et al. | <http://dx.doi.org/10.1007/s00134-014-3360-2> |
| Early ﬂuid resuscitation and volume therapy in venoarterial extracorporeal membrane oxygenation | 2016 | Journal of Critical Care | Staudacher, Gold, Biever et al. | <http://dx.doi.org/10.1016/j.jcrc.2016.09.017> |
| Early positive fluid balance is associated with mortality in patients treated with veno-arterial extracorporeal membrane oxygenation for cardiogenic shock: a retrospective cohort study | 2020 | Shock | Besnier, Boubèche, Clavier et al. | DOI: 10.1097/SHK.0000000000001381 |
| Executive Summary: Clinical Practice Guidelines  for the Prevention and Management of Pain,  Agitation/Sedation, Delirium, Immobility, and Sleep  Disruption in Adult Patients in the ICU | 2018 | Critical Care Medicine | Devlin, Skrobik, Gélinas et al. | DOI: 10.1097/CCM.0000000000003259 |

## Topic 5. Monitoring, Optimal balance blood pressure – vasoactive medication, “less is more?”

**Monitoring | Optimal balance blood pressure – vasoactive medication, “less is more?”**

P – Patients supported with VA ECMO

I – Accepting a lower mean arterial pressure (MAP<65 mmHg)

C – Standard MAP ≥65 mmHg

O – In-hospital mortality, LOS

Mesh-terms

- Extracorporeal membrane oxygenation
  - Extracorporeal membrane oxygenation/mortality
  - Extracorporeal membrane oxygenation/therapy
- Dose-Response Relationship, Drug
- Microcirculation
  - Microcirculation/drug effects
  - Microcirculation/therapy
- Dose-Response Relationship, Drug
- Vasoconstrictive agents
  - Vasoconstrictive agents/administration and dosage
  - Vasoconstrictive agents/drug therapy
  - Vasoconstrictive agents/standards
  - Vasoconstrictive agents/therapeutic use
  - Vasoconstrictive agents/therapy
- Hypotension
  - Hypotension/drug therapy
  - Hypotension/therapy

**Search: PubMed**

#1

( Extracorporeal Membrane Oxygenation/mortality[Mesh] OR Extracorporeal Membrane Oxygenation/therapeutic use[Mesh] OR Extracorporeal Membrane Oxygenation/therapy[Mesh] OR Venoarterial ECMO [tiab] OR VA ECMO [tiab])

**AND**

#2
(permissive hypotension [tiab] OR hypotensive resuscitation[tiab] OR mean arterial pressure[tiab] OR Hypotension/drug effects[Mesh]) OR Hypotension/therapy[Mesh] OR Hypotension [Mesh] OR MAP [tiab])

**NOT**

#3
(Pediatrics [Mesh] OR pediatric* [tiab] OR paediatric* [tiab] OR children*[tiab] OR neonatal [tiab] OR NICU [tiab])

**AND**

#4

( Microcirculation/drug effects[Mesh] OR Microcirculation/therapy[Mesh] OR dose-response relationship, drug [Mesh] OR noradrenaline[tiab] OR norepinephrine [tiab] OR vasoactive medic*[tiab] OR vasopressor*[tiab] OR inotrop*[tiab] OR Vasoconstrictor Agents/administration and dosage[Mesh] OR Vasoconstrictor Agents/drug therapy[Mesh] OR Vasoconstrictor Agents/standards[Mesh] OR Vasoconstrictor Agents/therapeutic use[Mesh] OR Vasoconstrictor Agents/therapy[Mesh] )

Results Pubmed
*#1 and #2 results in 74 hits
#1, #2 and #3 results in 58 hits
#1, #2 and #4 results in 16 hits
#1, #2, #3 and #4 results in 13 hits*

**Search: Web of Science**

Date run: 02-08-2022

#1

TS= Extracorporeal Membrane Oxygenation OR TS= Venoarterial ECMO OR TS = VA ECMO or TI= venoarterial ECMO or TI= VA ECMO OR AB= venoarterial ECMO OR AB = venoarterial ecmo

#2

TS =permissive hypotension OR TI = permissive hypotension OR AB=permissive hypotension OR ts = hypotensive resuscitation OR TI= hypotensive resuscitation OR AB= hypotensive resuscitation OR ts= mean arterial pressure OR TI = mean arterial pressure OR AB = mean arterial pressure OR TI=MAP OR AB = MAP

#3

TS = Pediatric* OR TI=pediatric* OR AB = pediatric OR TS =children* OR TI = children* OR AB=children* OR TS= neonatal OR TI= neonatal OR AB= neonatol OR TS= NICU or TI = NICU OR AB= NICU

#4

TS = Microcirculation OR TS= ''dose-response relationship'' OR TS =noradrenaline OR TI = noradrenaline OR AB = noradrenaline OR TS = norepinephrine OR TI = norepinephrine OR AB = norepinephrine OR TS = vasoactive medic* OR TI=vasoactive medic* OR AB = vasoactive medic OR TS = vasopressor* OR TI= vasopressor* OR AB= vasopressor* OR TS= inotrop* OR TI=inotrop* OR AB=inotrop*

Results Web of Science

*#1 and #2 results in 463 hits
#1, #2 and #3 results in 325 hits
#1, #2 and #4 results in 73 hits
#1, #2, #3 and #4 results in 50 hits*


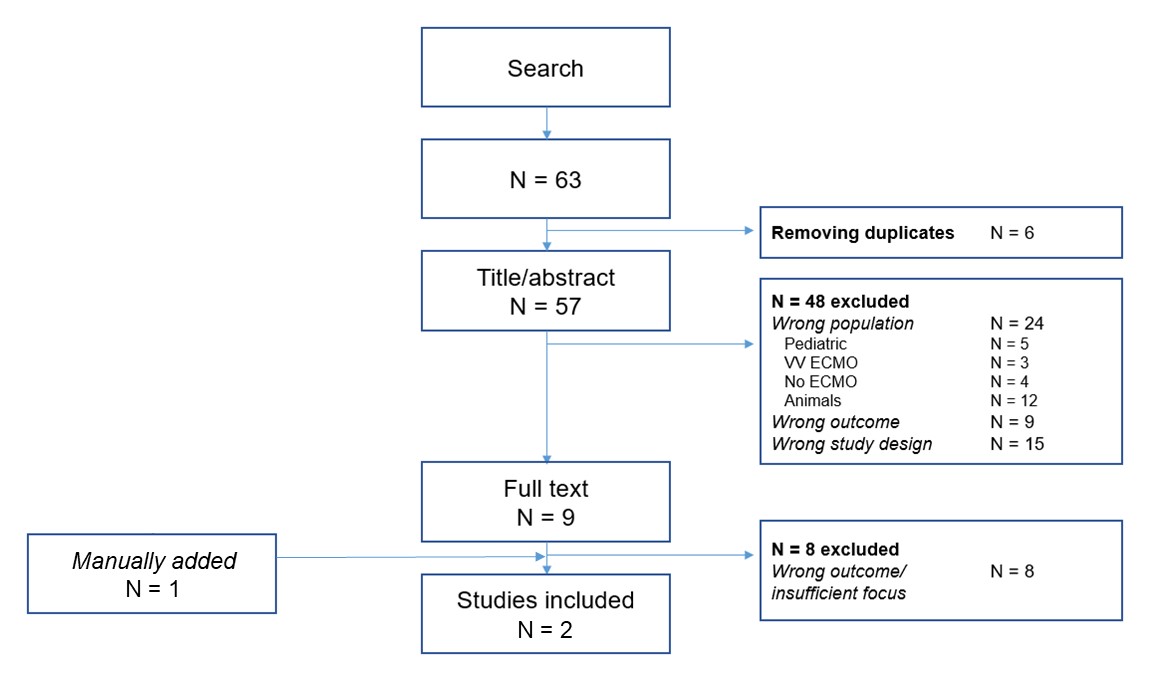


| **Title** | **Year** | **Journal** | **Authors** | **doi** |
| --- | --- | --- | --- | --- |
| What Is the Optimal Blood Pressure on Veno-Arterial Extracorporeal Membrane Oxygenation? Impact of Mean Arterial Pressure on Survival | 2019 | ASAIO | Tanaka D, Shimada S., Mullin M, Kreitler K, Cavarocchi N, Hirose H | 10.1097/mat.0000000000000824 |
| Blood-Pressure Targets in Comatose Survivors of Cardiac Arrest | 2022 | NEJM | Kjaergaard J, Möller J, Schmidt H et al. | 10.1056/NEJMoa2208687 |

## Topic 6. Blood transfusion regimen

**Adjuvant treatments | Blood transfusion regimen**

P – Patients supported with VA ECMO

I – Restrictive (threshold dependent on blood product)

C – Liberal (threshold dependent on blood product)

O – in-hospital mortality, LOS

*With special focus on the following sub-topics*

- Red blood cells
- Platelets
- Plasma

Mesh-terms

- Extracorporeal membrane oxygenation
  - Extracorporeal membrane oxygenation/mortality
  - Extracorporeal membrane oxygenation/therapy
- Blood Transfusion
  - Blood transfusion/methods*
- Platelet transfusion
  - Platelet transfusion/methods
  - Platelet transfusion/standards*
  - Platelet transfusion/statistics & numerical data
  - Platelet transfusion/therapeutic use
  - Platelet transfusion/therapy
- Erythrocyte transfusion
  - Erythrocyte transfusion/methods
  - Erythrocyte transfusion/standards*
  - Erythrocyte transfusion/statistics & numerical data
  - Erythrocyte transfusion/therapeutic use
  - Erythrocyte transfusion/therapy
- Adult
- Plasma*

**Search: PubMed**

#1

( "Extracorporeal Membrane Oxygenation/mortality"[Mesh] OR "Extracorporeal Membrane Oxygenation/therapeutic use"[Mesh] OR "Extracorporeal Membrane Oxygenation/therapy"[Mesh] OR “Venoarterial ECMO” [tiab] OR “VA ECMO” [tiab])

**AND**

#2
(“Threshold” [tiab] OR “regimen” [tiab] OR “restrictive”[tiab] OR “liberal” [tiab] OR “transfusion strategies” [tiab])

**AND**

#3
(“Red blood cell*” [tiab] OR “RBC” [tiab] OR “Erythrocyte*”[tiab] OR “Red cell transfusion” [tiab] OR "Erythrocyte Transfusion/methods"[Mesh] OR "Erythrocyte Transfusion/standards"[Mesh] OR "Erythrocyte Transfusion/statistics and numerical data"[Mesh] OR "Erythrocyte Transfusion/therapeutic use"[Mesh] OR "Erythrocyte Transfusion/therapy"[Mesh] )

**AND**

#4
(“Platelet*”[tiab] OR “Thrombocyte”[tiab] OR "Platelet Transfusion/methods"[Mesh] OR "Platelet Transfusion/standards"[Mesh] OR "Platelet Transfusion/statistics and numerical data"[Mesh] OR "Platelet Transfusion/therapeutic use"[Mesh] OR "Platelet Transfusion/therapy"[Mesh] )

**AND**

#5
(“Plasma”[Mesh] OR “Blood Transfusion” [Mesh] OR “Blood transfusion/methods*”[Mesh] OR “Fresh frozen plasma” [tiab] OR “convalescent plasma”[tiab] OR “plasma transfusion”[tiab] OR “FFP”[tiab])

**NOT**

#6
(“Pediatrics” [Mesh] OR “pediatric*” [tiab] OR “paediatric*” [tiab] OR “children*”[tiab] OR “neonatal” [tiab] OR “NICU” [tiab])

Results Pubmed

1. *#1 and #2 results in 63 hits*
2. *#1, #2 and #3 results in 9 hits*
3. *#1, #2 and #4 results in 9 hits*
4. *#1, #2 and #5 results in 13 hits*
5. *#1, #3 and #6 results in 85 hits*
6. *#1, #4 and #6 results in 160 hits*
7. *#1, #5 and #6 results in 91 hits*
8. *#1, #2, #3 and #6 results in 4 hits*
9. *#1, #2, #4 and #6 results in 4 hits*
10. *#1, #2, #5 and #6 results in 7 hits*

**Results from searches 1, 5, 6 and 7 combined**


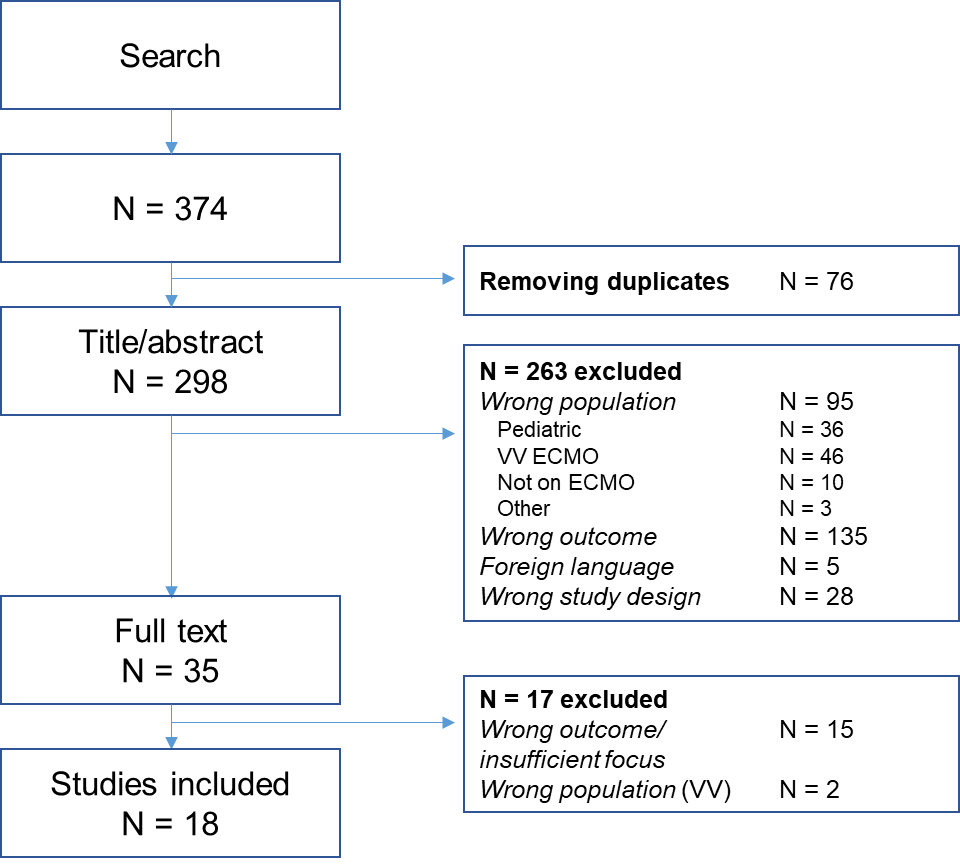


| **Title** | **Year** | **Journal** | **Authors** | **doi** |
| --- | --- | --- | --- | --- |
| Red blood cell transfusion requirements for patients on extracorporeal membrane oxygenation. | 2022 | Perfusion | Choi MH, Alvarez NH, Till BM, Tsypin Y, Sparks B, Hirose H | <https://doi.org/10.1177/0267659121998944> |
| Transfusion and its association with mortality in patients receiving veno-arterial extracorporeal membrane oxygenation. | 2022 | Journal of critical care | McCloskey CG, Engoren MC | <https://doi.org/10.1016/j.jcrc.2021.11.012> |
| Blood Transfusion Threshold in Patients Receiving Extracorporeal Membrane Oxygenation Support for Cardiac and Respiratory Failure-A Systematic Review and Meta-Analysis. | 2021 | Journal of cardiothoracic and vascular anesthesia | Abbasciano RG, Yusuff H, Vlaar APJ, Lai F, Murphy GJ | <https://doi.org/10.1053/j.jvca.2020.08.068> |
| Platelet Transfusion and In-Hospital Mortality in Veno-Arterial Extracorporeal Membrane Oxygenation Patients. | 2021 | ASAIO | Mazzeffi M, Rabin J, Deatrick K, Krause E, Madathil R, Grazioli A, Bathula A, Jackson B, Taylor B, Plazak M | <https://doi.org/10.1097/mat.0000000000001643> |
| Platelet transfusion is associated with 90-day and 1-year mortality for adult patients requiring veno-arterial extracorporeal membrane oxygenation. | 2021 | Vox sanguinis | Esper SA, Wallisch WJ 4th, Ryan J, Sanchez P, Sciortino C, Murray H, Arlia P, D'Cunha J, Mahajan A, Triulzi D, Subramaniam K | <https://doi.org/10.1111/vox.13016> |
| Platelets and extra-corporeal membrane oxygenation in adult patients: a systematic review and meta-analysis. | 2020 | Intensive care medicine | Jiritano F, Serraino GF, Ten Cate H, Fina D, Matteucci M, Mastroroberto P, Lorusso R | <https://doi.org/10.1007/s00134-020-06031-4> |
| Impact of massive blood transfusion during adult extracorporeal membrane oxygenation support on long-term outcomes: a nationwide cohort study in Taiwan. | 2020 | BMJ open | Chen FT, Chen SW, Wu VC, Hung KC, Chang SH, Ting PC, Chou AH | <https://doi.org/10.1136/bmjopen-2019-035486> |
| Multi-Modal Characterization of the Coagulopathy Associated With Extracorporeal Membrane Oxygenation. | 2020 | Critical care medicine | Granja T, Hohenstein K, Schüssel P, Fischer C, Prüfer T, Schibilsky D, Wendel HP, Jaschonek K, Serna-Higuita L, Schlensak C, Häberle H, Rosenberger P, Straub A | <https://doi.org/10.1097/ccm.0000000000004286> |
| Frequency of Thrombocytopenia and Heparin-Induced Thrombocytopenia in Patients Receiving Extracorporeal Membrane Oxygenation Compared With Cardiopulmonary Bypass and the Limited Sensitivity of Pretest Probability Score. | 2020 | Critical care medicine | Arachchillage DRJ, Laffan M, Khanna S, Venbriele C, Kamani F, Passariello M, Rosenberg A, Aw TC, Banya W, Ledot S, Patel BV | <https://doi.org/10.1097/ccm.0000000000004261> |
| Prognostic impact of blood product transfusion in VA and VV ECMO. | 2019 | Perfusion | Guimbretière G, Anselmi A, Roisne A, Lelong B, Corbineau H, Langanay T, Flaecher E, Verhoye JP | <https://doi.org/10.1177/0267659118814690> |
| Hemorrhagic complications during extracorporeal membrane oxygenation - The role of anticoagulation and platelets. | 2019 | Journal of critical care | Oude Lansink-Hartgring A, de Vries AJ, Droogh JM, van den Bergh WM | <https://doi.org/10.1016/j.jcrc.2019.09.013> |
| Implementation of a Standardized Transfusion Protocol for Cardiac Patients Treated With Venoarterial Extracorporeal Membrane Oxygenation Is Associated With Decreased Blood Component Utilization and May Improve Clinical Outcome. | 2018 | Anesthesia and analgesia | Cahill CM, Blumberg N, Schmidt AE, Knight PA, Melvin AL, Massey HT, Delehanty JM, Zebrak SB, Refaai MA | <https://doi.org/10.1213/ane.0000000000002238> |
| Blood Transfusion Strategies in Patients Undergoing Extracorporeal Membrane Oxygenation. | 2017 | Korean journal of critical care medicine | Kim HS, Park S | <https://doi.org/10.4266/kjccm.2016.00983> |
| Bleeding, Transfusion, and Mortality on Extracorporeal Life Support: ECLS Working Group on Thrombosis and Hemostasis. | 2016 | The Annals of thoracic surgery | Mazzeffi M, Greenwood J, Tanaka K, Menaker J, Rector R, Herr D, Kon Z, Lee J, Griffith B, Rajagopal K, Pham S | <https://doi.org/10.1016/j.athoracsur.2015.07.046> |
| Prognostic Impact of Persistent Thrombocytopenia During Extracorporeal Membrane Oxygenation: A Retrospective Analysis of Prospectively Collected Data From a Cohort of Patients With Left Ventricular Dysfunction After Cardiac Surgery. | 2016 | Critical care medicine | Opfermann P, Bevilacqua M, Felli A, Mouhieddine M, Bachleda T, Pichler T, Hiesmayr M, Zuckermann A, Dworschak M, Steinlechner B | <https://doi.org/10.1097/ccm.0000000000001964> |
| Extracorporeal membrane oxygenation support in acute coronary syndromes complicated by cardiogenic shock. | 2015 | Catheterization and cardiovascular interventions | Esper SA, Bermudez C, Dueweke EJ, Kormos R, Subramaniam K, Mulukutla S, Sappington P, Waters J, Kh,har SJ | <https://doi.org/10.1002/ccd.25871> |
| Factors associated with outcomes of patients on extracorporeal membrane oxygenation support: a 5-year cohort study. | 2013 | Critical care (London, England) | Aubron C, Cheng AC, Pilcher D, Leong T, Magrin G, Cooper DJ, Scheinkestel C, Pellegrino | <https://doi.org/10.1186/cc12681> |
| Blood transfusion requirements and independent predictors of increased transfusion requirements among adult patients on extracorporeal membrane oxygenation -- a single centre experience. | 2009 | Vox sanguinis | Ang AL, Teo D, Lim CH, Leou KK, Tien SL, Koh MB | <https://doi.org/10.1111/j.1423-0410.2008.01110.x> |

## Topic 7. Adjuvant treatments: anticoagulant therapy

**Adjuvant treatments | anticoagulant therapy**

P – Patients supported with VA ECMO

I – non-heparin anticoagulant therapy (*see subgroups*)

C – continuous intravenous heparin

O – in-hospital mortality, LOS

*With special focus on the following sub-topics*

- LMWH
- Bivalirudin
- DOAC

Mesh-terms

- Extracorporeal membrane oxygenation
  - Extracorporeal membrane oxygenation/adverse effects
  - Extracorporeal membrane oxygenation/mortality
  - Extracorporeal membrane oxygenation/therapy
- Anticoagulants
  - Anticoagulants/administration & dosage
  - Anticoagulants/blood
  - Anticoagulants/pharmacology
  - Anticoagulants/standards
  - Anticoagulants/statistics and numerical data
  - Anticoagulants/therapeutic use
  - Anticoagulants/therapy
- Heparin
- Heparin, Low-Molecular Weight
- Hirudins (bivalirudin)

**Search: PubMed (15-8-2022)**

#1

( "Extracorporeal Membrane Oxygenation/mortality"[Mesh] OR "Extracorporeal Membrane Oxygenation/therapeutic use"[Mesh] OR "Extracorporeal Membrane Oxygenation/adverse effects"[Mesh] OR "Extracorporeal Membrane Oxygenation/therapy"[Mesh] OR “Venoarterial ECMO” [tiab] OR “VA ECMO” [tiab])

**AND**

#2
( "Anticoagulants "[Mesh] OR "Anticoagulants/blood"[Mesh] OR "Anticoagulants/pharmacology"[Mesh] OR "Anticoagulants/standards"[Mesh] OR "Anticoagulants/statistics and numerical data"[Mesh] OR "Anticoagulants/therapeutic use"[Mesh] OR "Anticoagulants/therapy"[Mesh] OR “Anticoagul*” [tiab])

**NOT**

#3
(“Pediatrics” [Mesh] OR “pediatric*” [tiab] OR “paediatric*” [tiab] OR “children*”[tiab] OR “neonatal” [tiab] OR “NICU” [tiab])

**AND**

#4
(“Heparin”[Mesh] OR “heparin”[tiab])

**AND**

#5
( "Hirudins/administration and dosage"[Mesh] OR "Hirudins/blood"[Mesh] OR "Hirudins/pharmacology"[Mesh] OR "Hirudins/standards"[Mesh] OR "Hirudins/therapeutic use"[Mesh] OR “Hirudins” [Mesh] OR “bivalirudin”[tiab] OR “direct thrombin inhibitor”[tiab] OR “DTI”[tiab] OR “Angiomax”[tiab] OR “Angiox”[tiab])

**AND**

#6
(“Heparin, Low-Molecular-Weight”[Mesh] OR "Heparin, Low-Molecular-Weight/administration and dosage"[Mesh] OR "Heparin, Low-Molecular-Weight/blood"[Mesh] OR "Heparin, Low-Molecular-Weight/pharmacology"[Mesh] OR "Heparin, Low-Molecular-Weight/standards"[Mesh] OR "Heparin, Low-Molecular-Weight/statistics and numerical data"[Mesh] OR "Heparin, Low-Molecular-Weight/therapeutic use"[Mesh] OR “LMWH” [tiab] OR “Low Molecular Weight Heparin”[tiab])

Results PubMed
*#1 and #2 results in 387 hits
#1, #2 and #3 results in 289 hits
#1, #2, #3 and #4 results in 142 hits
#1, #2, #3 and #5 results in 15 hits
#1, #2, #3 and #6 results in 8 hits*


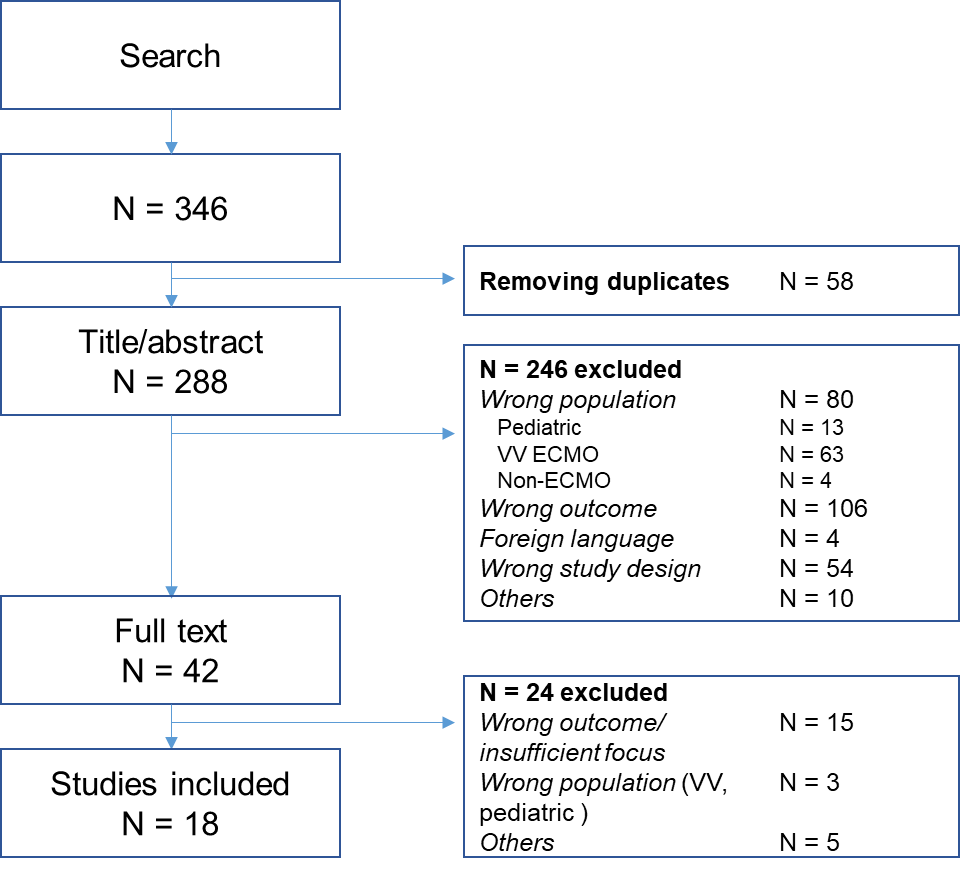


| **Title** | **Year** | **Journal** | **Authors** | **doi** |
| --- | --- | --- | --- | --- |
| Low-dose heparin during extracorporeal membrane oxygenation treatment in adults. | 2015 | Intensive Care Medicine | Yeo, Kim, Jeon et al. |  |
| Low-Dose Versus Therapeutic Anticoagulation in Patients on Extracorporeal Membrane Oxygenation: A Pilot Randomized Trial. | 2019 | Critical Care Medicine | Aubron, McQuilten, Bailey et al. |  |
| A comparison of low and standard anti-coagulation regimens in extracorporeal membrane oxygenation. | 2019 | J Heart Lung Transplant | Raman, Alimohamed, Dobrilovic et al. | https://doi.org/10.1016/j.healun.2019.01.1313 |
| Enoxaparin vs. unfractionated heparin for anticoagulation during continuous veno-venous hemofiltration: A randomized controlled crossover study. | 2007 | Intensive Care Medicine | Joannidis, Kountchev, Rauchenzauner et al. |  |
| Low molecular weight heparin versus unfractioned heparin for anticoagulation during perioperative extracorporeal membrane oxygenation: A single center experience in 102 lung transplant patients. | 2020 | Artifical Organs | Gratz, Pausch, Schaden et al. |  |
| Argatroban Anticoagulation for Adult Extracorporeal Membrane Oxygenation: A Systematic Review | 2022 | Journal of Intensive Care Medicine | Geli, Capoccia, Maybauer et al. |  |
| Extracorporeal Membrane Oxygenation Complications in Heparin- and Bivalirudin-Treated Patients | 2021 | Critical Care Explor. | Giuliano, Bielow, Etchill et al. |  |
| Comparison of Bivalirudin Versus Unfractionated Heparin for Anticoagulation in Adult Patients on Extracorporeal Membrane Oxygenation | 2022 | ASAIO Journal | Sheridan, Sekala, Pandya et al. |  |
| Venoarterial-Extracorporeal Membrane Oxygenation Without Routine Systemic Anticoagulation Decreases Adverse Events. | 2020 | Annals of Thoracic Surgery | Wood, Ayers, Gosev et al. |  |
| Reduced anticoagulation targets in extracorporeal life support (RATE): study protocol for a randomized controlled trial | 2022 | Trials | van Minnen, Oude Lansink- Hartgring, van den Boogaard et al. |  |

## Topic 8. Complications: endothelial activation and damage

**Complications | Endothelial activation and damage *Suggested authors: Carolien Volleman, Charissa van den Brom***
P – Patients supported with VA ECMO
I – Measuring ICAM-1, VCAM, Syndecan-1 to monitor endothelial activation and damage
C – No additional measurements
O – Mortality, LOS (outcome can still change)

Mesh terms

- Extracorporeal membrane oxygenation
- Endothelium, vascular
- Glycocalyx
- Intercellular Adhesion Molecule-1
- Vascular Cell Adhesion Molecule-1
- Syndecans
- Soluble Thrombomodulin
- Von Willebrand Factor
- Angiopoietin-2

**Search: PubMed**

#1
("Extracorporeal Membrane Oxygenation" [Mesh] OR “extracorporeal membrane oxygenation” [tiab] OR “Venoarterial ECMO” [tiab] OR “VA ECMO” [tiab] OR “ECPR” [tiab])

**AND**

#2

("Endothelium, Vascular" [Mesh] OR “endothelium” [tiab] OR “endothelial” [tiab] OR “endothelial activation” [tiab] OR “endothelial damage” [tiab] OR “glycocalyx” [Mesh] OR “glycocalyx” [tiab])

**AND/OR**

#3

("Intercellular Adhesion Molecule-1" [Mesh] OR “ICAM-1” [tiab] OR “ICAM” [tiab] OR “Intercellular Adhesion Molecule” [tiab] OR "Vascular Cell Adhesion Molecule-1" [Mesh] OR “VCAM” [tiab] OR “Vascular Adhesion Molecule” [tiab] OR "Syndecans" [Mesh] OR “syndecan” [tiab] OR “thrombomodulin” [ Mesh] or “von Willebrand factor” [Mesh] OR “Angiopoietin-2” [Mesh])

**AND**

#4

("Length of Stay" [Mesh] OR “length of stay” [tiab] OR “length-of-stay” [tiab] OR “hospital stay” [tiab] OR “hospital duration” [tiab])

Results Pubmed
*#1 and #2 results in 133 hits*

*#1 and #3 results in 34 hits*

*#1 and #2 and #3 results in 12 hits*

*#1 and #2 OR #3 results in 155 hits*

*#1 and #2 OR #3 AND #4 results in 3 hits*

**Search: Embase**

**#1**

'extracorporeal oxygenation':ti,ab OR 'ecmo':ti,ab OR 'veno-arterial ecmo':ti,ab

**AND**

**#2**

'endothelium':ti,ab OR 'endothelial dysfunction':ti,ab OR 'endothelial activation':ti,ab OR 'glycocalyx':ti,ab

**AND/OR**

**#3**

'intracellular adhesion molecule 1'/exp OR 'intracellular adhesion molecule 1':ti,ab OR 'vascular cell adhesion molecule 1'/exp OR 'vascular cell adhesion molecule 1':ti,ab OR 'syndecan'/exp OR 'syndecan':ti,ab OR 'angiopoietin-2'/exp OR 'angiopoietin-2':ti,ab OR 'von willebrand factor'/exp OR 'von willebrand factor':ti,ab OR 'thrombomodulin'/exp OR 'thrombomodulin':ti,ab

Results Embase
*#1 and #2 results in 62 hits*

*#1 and #3 results in 136 hits*

*#1 and #2 and #3 results in 13 hits*

*#1 and #2 OR #3 results in 185 hits*

**Search: Web of Science**

#1
**(((((TI=(ECMO)) OR AB=(ECMO)) OR TI=(extracorporeal membrane oxygenation)) OR AB=(extracorporeal membrane oxygenation)) OR TI=(veno-arterial ECMO)) OR AB=(veno-arterial ECMO)**

AND

#2
**((((((((( TI=(endothelium)) OR AB=(endothelium)) OR TI=(endothelial dysfunction)) OR AB=(endothelial dysfunction)) OR TI=(endothelial activation)) OR AB=(endothelial activation)) OR TI=(endothelial damage)) OR AB=(endothelial damage)) OR TI=(glycocalyx)) OR AB=(glycocalyx)**

AND/OR

#3
**((((((((((((((( TI=(intracellular adhesion molecule)) OR AB=(intracellular adhesion molecule)) OR TI=(ICAM)) OR AB=(ICAM)) OR TI=(vascular cell adhesion molecule)) OR AB=(vascular cell adhesion molecule)) OR TI=(VCAM)) OR AB=(VCAM)) OR TI=(syndecan)) OR AB=(syndecan)) OR TI=(angiopoietin)) OR AB=(angiopoietin)) OR TI=(von willebrand factor)) OR AB=(von willebrand factor)) OR TI=(thrombomodulin)) OR AB=(thrombomodulin)**

Results Web of Science
*#1 and #2 results in 67 hits*

*#1 and #3 results in 71 hits*

*#1 and #2 and #3 results in 14 hits*

*#1 and #2 OR #3 results in 124 hits*

***
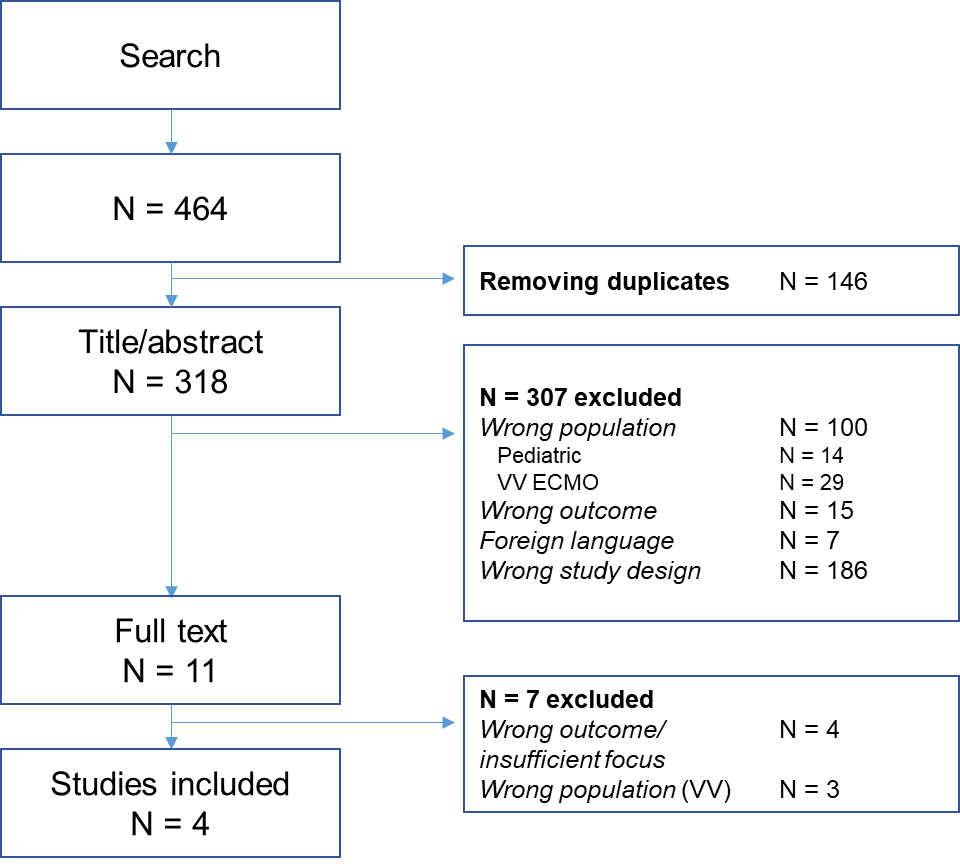
***

| **Title** | **Year** | **Journal** | **Authors** | **doi** |
| --- | --- | --- | --- | --- |
| Annexin V positive microvesicles are elevated and correlate with flow rate in patients receiving veno-arterial extracorporeal membrane oxygenation | 2020 | Interactive Cardiovascular and Thoracic Surgery | Siegel PM, Hentschel D, Bojti I, Wengenmayer T, Helbing T, Moser M, Duerschmied D, Trummer G, Bode C, Diehl P | <https://doi.org/10.1093/icvts/ivaa198> |
| Prognostic value of endothelial biomarkers in refractory cardiogenic shock with ECLS: a prospective monocentric study | 2019 | BMC Anesthesiology | Tsai TY, Tu KH, Tsai FC, Nan YY, Fan PC, Chang CH, Tian YC, Fang JT, Yang CW, Chen YC. | <https://doi.org/10.1186/s12871-019-0747-1> |
| Extracellular Vesicles Are Associated With Outcome in Veno-Arterial Extracorporeal Membrane Oxygenation and Myocardial Infarction | 2021 | Front Cardiovasc Med | Siegel PM, Bender I, Chalupsky J, Heger LA, Rieder M, Trummer G, Wengenmayer T, Duerschmied D, Bode C, Diehl P. | <https://doi.org/10.3389/fcvm.2021.747453> |
| Von Willebrand Factor-GP1ba Interactions in Venoarterial Extracorporeal Membrane Oxygenation Patients | 2019 | J Cardiothorac Vasc Anesth | Mazzeffi M, Hasan S, Abuelkasem E, Meyer M, Deatrick K, Taylor B, Kon Z, Herr D, Tanaka K. | <https://doi.org/10.1053/j.jvca.2018.11.031> |
